# Biohybrid Vascular Graft Made of Textile‐Reinforced Elastin‐Like Recombinamers and Its Preservation via Drying Processes

**DOI:** 10.1002/adhm.202500482

**Published:** 2025-05-03

**Authors:** Dominic Pascal Andre, Stephan Ruetten, José Carlos Rodríguez‐Cabello, Stefan Jockenhoevel, Thomas Schmitz‐Rode, Alicia Fernández‐Colino

**Affiliations:** ^1^ Department of Biohybrid and Medical Textiles (BioTex) AME‐Institute of Applied Medical Engineering Helmholtz Institute RWTH Aachen University 52074 Aachen Germany; ^2^ Electron Microscopy Facility Uniklinik RWTH Aachen 52074 Aachen Germany; ^3^ Bioforge Lab Group for Advanced Materials and Nanobiotechnology Biomedical Networking Research Center of Bioengineering Biomaterials and Nanomedicine (CIBER‐BBN) Edificio LUCIA Universidad de Valladolid Valladolid 47011 Spain; ^4^ AME‐Institute of Applied Medical Engineering Helmholtz Institute RWTH Aachen University 52074 Aachen Germany

**Keywords:** drying, engineered elastin, textile, translation, vascular graft

## Abstract

Vascular grafts are crucial for treating cardiovascular diseases and providing vascular access for hemodialysis in end‐stage renal disease, conditions that affect millions of people globally. To address the persisting clinical need for better therapy for these conditions, new designs involving novel materials and innovative tissue‐engineered approaches are being developed. Successful clinical translation of such designs will require to ensure device safety, particularly sterility and mechanical integrity. The prevailing method for ensuring sterility is ethylene oxide sterilization, which requires a dry product. The challenge of drying biohybrid implants is substantial, as they contain multiple components (e.g., textile and hydrogel) with differing properties. To address this open question, the effects of different drying methods on the morphological and mechanical properties of biohybrid implants made from elastin‐like recombinamers (ELRs) are investigated. For that, mechanical characteristics defined in ISO 7198, as well as the cell attachment behavior on biohybrid vascular grafts, treated either with lyophilization (LYO) or CO_2_‐based critical point drying, are compared. The results show that the applied drying method can significantly influence the properties of the scaffolds and highlight the importance of developing implant‐specific drying schemes that ensure its safety and functionality.

## Graphical abstract

Clinical translation of biohybrid medical implants requires the previous implementation of drying schemes that can facilitate their sterilization and storage. This study addresses that critical aspect and elucidates the impact of drying on biohybrid vascular grafts, highlighting the need for implant‐specific drying strategies to ensure safety, functionality, and biocompatibility, in accordance with ISO 7198 standards.

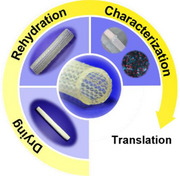

## 1 Introduction

Vascular grafts represent one of the main treatment options for many patients suffering cardiovascular disease and play a crucial role in providing and maintaining vascular access for hemodialysis in patients suffering from end stage renal disease.^[^ ^1^, ^2^ ^]^ Currently available artificial grafts are typically composed of synthetic polymers and unfortunately, have inadequate compliance, hemocompatibility, biocompatibility, and no self‐healing properties, leading to many complications like intima hyperplasia, thrombus, and pseudoaneurysm formation.^[^ ^1^, ^3^, ^4^, ^5^, ^6^, ^7^, ^8^, ^9^ ^]^ Despite these mentioned complications, synthetic grafts have proven effective in the treatment of conditions affecting larger diameter (>6 mm) blood vessels. However, small‐diameter (<6 mm) artificial grafts are still prone to prosthesis failure due to thrombosis, because of the high ratio of blood‐contacting artificial surface area to blood volume, as well as the low flow conditions present in small vessels.^[^ ^10^ ^]^ Autologous vascular grafts still remain the gold standard for small diameter blood vessel replacement due to their higher patency rates compared to alternative artificial solutions.^[^ ^11^, ^12^ ^]^ However, the use of autologous vessels is constrained by several factors, including limited availability, associated donor site morbidity, and often poor quality as a consequence of underlying medical conditions, like arteriosclerosis or diabetes type 2.^[^ ^13^, ^14^ ^]^ Therefore, there is a persisting clinical need for better artificial vascular grafts allowing for efficient and safe long‐term functionality. To address this issue, research groups are exploring alternative cell‐based concepts (e.g., following the principles of classical tissue engineering) or material‐centric approaches (i.e., following in situ tissue engineering schemes).^[^ ^15^, ^16^, ^17^, ^18^, ^19^ ^]^ The clinical translation process for cell‐based concepts of tissue engineered vascular grafts faces significant hurdles, such as ensuring consistent quality with high donor‐dependent variability in cell behavior and long production times leading to high production costs. The living nature of these concepts complicates their storage and therefore also their prompt availability, negatively impacting patients’ quality of life. These difficulties have propelled a focus shift from cell‐based toward cell‐free grafts, as a solution for faster potential clinical adoption.^[^ ^20^ ^]^ Culture‐free and cell‐free graft production allows for off‐the‐shelf availability, faster fabrication, potentially higher reproducibility and, through that, a reduced regulatory burden.^[^ ^21^ ^]^ Textile‐reinforced hydrogels are emerging as an appealing approach to engineer biohybrid cell‐free and culture‐free implants. The biohybrid design is inspired by the structure of natural blood vessels, which can be regarded as a complex composite material.^[^ ^22^ ^]^ This natural structure consists of a hydrated matrix that is strengthened by an intricately arranged network of fibers. The fibers are organized in a directionally dependent (anisotropic) manner, providing specific mechanical properties to the vessel wall. Inspired by this natural architecture, the biohybrid design aims to replicate the functional characteristics of native blood vessels. In those concepts, a well‐designed textile scaffold guarantees long‐term stability in vivo, while the bio‐based hydrogel matrix provides an adequate microenvironment for in situ cell‐mediated remodeling.^[^ ^20^, ^21^, ^23^ ^]^

The unique combination of mechanically strong textile fibers and an elastic hydrogel matrix opens the possibility to mimic the behavior of native tissues.^[^ ^22^ ^]^ We have previously implemented such a concept for the fabrication of small caliber compliant vascular grafts using a macroporous matrix, made out of elastin‐like recombinamers (ELRs), reinforced with a warp‐knitted polyvinylidene fluoride (PVDF) mesh as well as an electrospun (e‐spun) layer for compliance regulation.^[^ ^20^ ^]^ In this concept, injection molding in combination with salt leaching/gas foaming was used to embed the PVDF mesh inside a macroporous ELR matrix, after which a polycaprolactone (PCL) layer of variable thickness was applied to the tubular scaffold via electrospinning. An RGD sequence was integrated into the ELR sequence as an integrin binding site to promote cell interaction. The created grafts exhibited native‐like mechanical properties, while additionally supporting cell ingrowth and the formation of a confluent endothelial layer.^[^ ^20^ ^]^ One important factor that enabled this approach is the high elasticity of ELRs in aqueous environment at body temperature. This elasticity stems from the entropically favorable conformation, characterized by an increase in type II β‐turns, which ELRs acquire above their transition temperature (*T*_t_). The driving force behind the elasticity of ELRs is therefore the return to this entropically favorable state. At temperatures below *T*_t_, ELRs are in extended (random coil) form. For ELR‐based hydrogels, this extended structure results in increased swelling and reduced elasticity compared to their state above *T*_t_.^[^ ^24^, ^25^, ^26^ ^]^

The progress done so far in the field of biohybrid medical implants is encouraging, and it will not be long till these textile‐reinforced concepts advance toward clinical translation, but for this to become a reality, there are still a few hurdles to overcome.^[^ ^22^ ^]^ First, a high level of reproducibility and standardization throughout the whole production process is required. It is also essential that the production of the vascular graft is fast, and easily scalable. Complex and time consuming production processes inevitably reduce the economic feasibility of a product and present one of the biggest hurdles for the clinical translation of innovative implant technologies.^[^ ^27^ ^]^ Aside from time‐efficient production, the finished product should also possess a long shelf life, must be easy to transport and store to facilitate availability and ensure the safety of a product. Ensuring that biomaterials remain sterile and free of contamination during storage is critical but challenging, as implants are susceptible to bacterial colonization and biofilm formation. Dry storage of biomaterials helps to slow down or prevent their deterioration, ensuring their intended properties and functionality are preserved.^[^ ^28^, ^29^, ^30^, ^31^ ^]^ A dry prothesis also allows for the application of a wider range of sterilization techniques, while guaranteeing an effective, reproducible and safe process.^[^ ^32^ ^]^ Unfortunately, drying a hydrogel can already lead to changes of the physical, mechanical, and biological characteristics of the material, like changes in the microstructure or material degradation, leading to a decrease in mechanical stability^[^ ^33^ ^]^ and biological function. The challenge of drying biohybrid implants is even higher, considering that such devices contain multiple components (textile and hydrogel) with differing properties, and therefore potentially differing responses to the same treatment.

Identifying and implementing a suitable drying scheme is therefore essential to the successful translation of textile‐reinforced hydrogel scaffolds. To address this open question, we investigated the effects of drying methods, commonly used in the post processing of biomaterials, on the physical and mechanical properties of biohybrid implants. The applied drying methods were lyophilization (LYO) as well as two different critical point drying (CPD) approaches utilizing either ethanol (ECPD) or acetone (ACPD) as the intermediate fluid for dehydration. LYO was chosen to evaluate how the potential introduction of porosity through ice crystal formation before drying, affects the characteristic of the graft.^[^ ^34^ ^]^ CPD on the other hand is a commonly used technique when preparing samples for scanning electron microscopy (SEM), that enables to obtain dried samples with little or no artifacts due to its limited effects on the microstructure.^[^ ^35^ ^]^ The goal of our work was to evaluate the impact of the drying methods on the mechanical and biological performance of the textile‐reinforced vascular graft, with prospects of implementing the necessary steps for implant storage, sterilization, and overall future translation. To this end, the mechanical characteristics defined in ISO 7198, as well as the rehydratability and bioactivity of biohybrid vascular grafts dried by the aforementioned methods, were compared.

## 2 Experimental Section

### 2.1 Graft Production

For the fabrication of the vascular grafts, two different ELRs, namely, DRIR and HRGD6, which crosslink with each other via catalyst free click‐chemistry to form a hydrogel, were used. The ELR called DRIR is a structural recombinamer with the proteolytic cleavage sequence Asp–Arg–Ile–Arg (DRIR),^[^ ^36^ ^]^ allowing for cell‐based remodeling of the scaffold after implantation. HRGD6 is a recombinamer containing an Arg–Gly–Asp (RGD) adhesion sequence, to facilitate cell attachment on the scaffold. The lysine residues of each ELR were chemically modified as previously reported,^[^ ^37^ ^]^ to introduce cyclooctyne and azide groups, resulting in DRIR‐cyclooctyne and HRGD6‐azide, respectively. The hydrogel was reinforced by a warp‐knitted polyethylene terephthalate (PET) textile. This choice was in response to the ongoing discussions about the health concerns and potential ban of fluorinated polymers as well as the fact that PET textiles are widely available as medical grade thanks to their wide usage in vascular implants.^[^ ^38^ ^]^ For the molding, each ELR component (100 mg mL^−1^) was dissolved for 1 h in a mixture (1:1 (v/v)) of phosphate buffer solution (PBS) (Gibco, pH: 7.4, Life Technologies, Carlsbad, CA, USA) and ethanol (Supelco, EMSURE, Sigma‐Aldrich, St. Louis, MO, USA). The two solutions were sequentially loaded into the same syringe and mixed by quickly inverting the syringe 20 times before being injected into the mold. The mold was constructed using a custom‐made outer shell made from polycarbonate (inner diameter = 9 mm) and a polished stainless steel inner core cylinder (outer diameter = 6 mm) placed coaxially to the shell. The components were joined together and sealed by a baseplate made from polyoxymethylene (POM) containing a silicone sealing ring. The heat set warp‐knitted polyethylene terephthalate textile was positioned concentrically in the annular space between the core cylinder and the outer shell. The PET‐mesh (6 mm diameter tricot, 1 × 1 lapping, EAC 10) was produced in‐house on a double needle bar raschel machine (MiniTronic 800, Rius‐Comatex, Barcelona, Spain). It was draped on a stainless‐steel hollow core (outer diameter = 7.5 mm), with both ends fixed by clamps, and subsequentially heat set by autoclaving at 121 °C for 20 min. During the molding, the space was filled with the ELR mixture. After incubating for 30 min at room temperature, the shell was opened, and the ELR‐textile scaffold carefully removed from the core before being placed in an ethanol solution (70% (v/v) until further treatment or characterization. Before characterization all scaffolds were washed and incubated in PBS for 3 × 30 min followed by 1 × 1.5 h at room temperature.

### 2.2 Drying Methods

**Figure 1:**
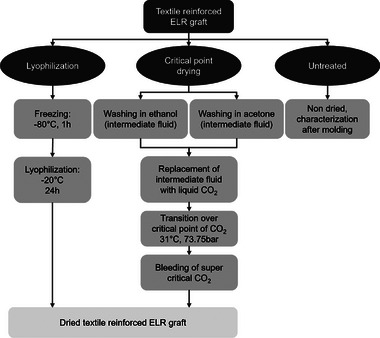
Schematic overview of the different treatments. Lyophilization (LYO) after step‐down freezing at −80 °C and critical‐point drying (CPD) in CO_2_ after immersion in either absolute ethanol (ECPD) or absolute acetone (ACPD).

Three different drying methods were evaluated, namely, LYO after step down freezing at ‐ 80 °C and CPD, with two different solvents as intermediate fluids (**Figure** **1**). These methods were chosen, as they are drying methods commonly used in the processing of hydrogel structures.

For LYO, the grafts were prepared as described above, then all excess PBS was removed using laboratory wipes (KIMBERLY‐CLARK GmbH, Koblenz, Germany) before being placed in a −80 °C freezer for 1 h. They were then lyophilized for 24 h at −20 °C and a pressure of 0.050 mbar, using an Alpha 2–4 LSCPlus (Martin Christ Gefriertrocknungsanlagen GmbH, Osterode am Harz, Germany).

For the CPD, two approaches were used. In the first one, the samples were dehydrated by ethanol washing in a graded series of ethanol solutions with increasing concentration (40% (v/v) to 3 × 100%, in 10% increments, for 15 min in each solution). The second group of samples was dehydrated using a graded series of acetone solutions with the same parameters. The subsequent drying process was identical for both groups and performed using an E3100 critical point dryer (Quorum Technologies, Laughton, UK). The starting temperature was 14.5 °C when first flushing the chamber with liquid CO_2_ for 15 min. Sequenced purging every 10 min followed, with the pressure inside the device kept between 50 and 90 bar until all acetone/ethanol was removed from the chamber. The chamber was then vented until the CO_2_ phase boundary was right above the sample holder. At this point the temperature was increased to 37 °C to pass the critical point_._ Once the phase boundary vanished, the top valve was carefully opened, to start the bleeding of the CO_2_. Once atmospheric pressure was reached, the samples were removed and stored in dry state until further characterization.

### 2.3 Morphological Characterization

In order to conduct a systematic investigation of the effect of the applied drying method on the characteristics of textile‐reinforced hydrogel scaffold, the first step was to verify that the graft fabrication method led to reproducible results. Therefore, each graft was inspected right after production. The dimensions and appearance of the produced grafts were recorded to allow for an assessment of the production method. Specifically, the wall thickness, the inner diameter, and the textile positioning inside the hydrogel were evaluated. All treated grafts were further characterized after they were rehydrated in PBS (Gibco, pH: 7.4, Life Technologies, Carlsbad, CA, USA) at room temperature for 3 h, to recreate the implantable state of the prothesis.

#### 2.3.1 Wall Thickness and Relaxed Inner Diameter

The relaxed inner diameter of the prothesis was determined through indirect measurement. For that, 10 mm pieces were cut from differently treated grafts (*n* = 3 per condition) which were then placed for 10 min in PBS (Gibco, pH: 7.4, Life Technologies, Carlsbad, CA, USA) heated to 37 °C before measurement. The inner circumference of the graft was measured using a Keyence VHX 5000 Microscope (KEYENCE DEUTSCHLAND GmbH, Neu‐Isenburg, Germany) and their proprietary software, and from this circumference the inner diameter was calculated. The circumference was measured on both sides of each piece. The wall thickness of the prothesis was measured using the same pieces as for the diameter. For wall thickness, measurements at 10 equidistant points at each cutting surface were taken to calculate the mean wall thickness and standard deviation (SD).

#### 2.3.2 Quantification of Weight

For each treatment group, samples of 10 mm were cut from grafts (*n* = 3). For the evaluation of the weight in wet state, the samples were placed in PBS (Gibco, pH: 7.4, Life Technologies, Carlsbad, CA, USA) heated to 37 °C for 10 min prior to the measurement. All excess liquid was removed using KIMTECH SCIENCE laboratory wipes (KIMBERLY‐CLARK GmbH, Koblenz, Germany) after which the pieces were placed on an analytical balance Sartorius LA 120 S (Sartorius Lab Instruments GmbH & Co. KG, Goettingen, Germany). The weight was measured after production, drying, and rehydration. To check if full rehydration was reached, the weight was measured after 1, 2, and 3 h of rehydration in PBS at room temperature.

#### 2.3.3 Evaluation of Porosity by SEM

Cross‐sectional cuts of the grafts after drying them by either LYO, ACPD or ECPD were performed by submerging them in liquid nitrogen for 15 min and then breaking them, to minimize the chance for artifacts created by cutting with a blade. The cut samples were then mounted on aluminum stubs, with the cross‐section facing upward. For the sputter‐coating, a 20 nm layer of gold–palladium was applied, and the images were recorded with a Quattro S microscope (Thermo Fisher Scientific, Waltham, MA, USA) set to an accelerating voltage of 10 kV. The pore size distribution was then quantified using ImageJ software.^[^ ^39^ ^]^ For that measurements (*n* = 50) were taken from different samples (*n* = 3) to calculate the average pore size as well as the SD. To facilitate the identification of different regions, SEM pictures were colored using the Mountains software courtesy of Digital Surf (France).

### 2.4 Mechanical Characterization

#### 2.4.1 Tensile Testing

Cyclic tensile tests were conducted in order to assess the influence that the drying method has on the elastic behavior of the prothesis. The same samples that were previously used for the characterization of the graft dimensions were used in these tests. For each condition, three samples submerged in PBS (Gibco, pH: 7.4, Life Technologies, Carlsbad, CA, USA) at 37 °C, were tested. All samples were submerged in the heated PBS for 10 min prior to testing, to ensure equilibration to the target temperature. The tensile testing was performed in two steps using custom‐made sample holders, allowing for clamp free mounting of the ring samples in accordance with ISO 7198, connected to a 10 N load cell of a UniVert uniaxial tensile tester (CellScale biomaterials testing; Waterloo, Canada). The first step was a force controlled cyclic test in which the ring‐shaped samples were subjected to 200 cycles of loads equivalent to an internal pressure range from 30 to 170 mmHg. The required force was calculated using the following formula^[^ ^19^ ^]^

(1) $$F=d_{m}\;\times p_{\mathrm{mmHg}}\times L_{s}\left[N\right]$$

wherein *d*_m_ is the mean diameter of the ring in m, *p*_mmHg_ is the target pressure in Pa, and *L*_s_ is the initial sample length in m. *L*_s_ of each tested sample was calculated from measurements at three points using the Keyence VHX 5000 Microscope (KEYENCE DEUTSCHLAND GmbH, Neu‐Isenburg, Germany) and their proprietary software. The dilatation of the sample was assessed by determining the shift of the stress/strain curve from first cycle after preconditioning (cycle 5) to the cycle in which a plateau was reached for all treatment groups (cycle 150). The mean strain‐shift (*n* = 3 per condition) was calculated using the strain at 50% of the maximum stress. Afterward, a displacement‐controlled break test was conducted, using a 100 N load cell and a stretch rate of 50 mm min^−1^, with the maximum break strength being defined as the highest value reached before breaking of the sample occurred. The force–displacement curves were recorded to calculate the Young's modulus of the graft material, as well as to evaluate the stretching behavior of the material.

The same series of tensile tests were also performed on nonreinforced ELR samples to gain further insight into which component (textile or ELR matrix) is the main contributor to this shift behavior.

#### 2.4.2 Suture Retention

Suture retention strength was measured using two different sizes of suture material, namely, 6‐0 prolene and 7‐0 prolene. The suture was placed 2 mm from the edge of each tubular graft sample. The suture was fixed on the stationary clamp, connected to a 10 N load cell of a UniVert uniaxial tensile tester (CellScale biomaterials testing; Waterloo, Canada) and the grafts were clamped on the moving arm using custom‐made clamps to fulfill ISO requirements.^[^ ^40^ ^]^ The graft was fully immersed in PBS (Gibco, pH: 7.4, Life Technologies, Carlsbad, CA, USA), heated to 37 °C, for 10 min and then moved at a speed of 50 mm min^−1^ (in accordance with ISO guidelines) to pull the suture away from the graft. The force at break was recorded in Newton. For each treatment group measurements at four different points, located at 90° intervals along the edge, were conducted.

#### 2.4.3 Radial Compliance and Pressurized Inner Diameter

The grafts (*n* = 3 per condition, molded diameter 6 mm) were cut to a length of 6 cm, in accordance with ISO 7198 stating that the sample length must be at least 10× the inner diameter of the sample.^[^ ^40^ ^]^ The vascular grafts were mounted under 10% longitudinal stretch (to imitate the most likely in vivo conformation^[^ ^41^ ^]^) in a custom‐made bioreactor system previously described.^[^ ^20^ ^]^ The bioreactor included a chamber made of POM (Licharz GmbH, Buchholz, Germany) with clear poly(methyl methacrylate) sides and an optical micrometer model LS‐7030(M) (Keyence Deutschland GmbH, Neu‐Isenburg, Germany) to measure the outer diameter of the vascular graft by registering the size of the shadow on the sensor positioned across a parallel green laser from by emitter. Additionally, the system featured silicone tubes (Ismatec), an adjustable resistance to control the flow/pressure within the system, one in flow pressure sensor before and after the graft (Xtrans, Codan pvb Medical GmbH, Germany), and a small centrifugal pump (702‐6882, RS components, Corby, UK) controlled via a custom‐developed control unit.^[^ ^42^ ^]^ The flow‐loop was filled with PBS (Gibco, pH: 7.4, Life Technologies, Carlsbad, CA, USA) heated at 37 °C. The mean pressure was adjusted through modulating the pulse‐free flow by changing the power delivered to the centrifugal pump, in combination with the adjustable resistance. The pressure pulses (20/60, 30/70, 40/80, 50/90, 60/100, 80/120, 110/150 mmHg) were created, by changing the constant input voltage, using a sinusoidal function. The applied pressures and corresponding graft diameters were recorded with a custom‐developed LabVIEW program (LabVIEW 20.1, National Instruments). The average values of the compliance were calculated using the following formula

(2) $$C=\frac{\left(\frac{D_{2}-D_{1}}{D_{1}}\;\times \;10^{4}\right)}{P_{2}-P_{1}}\;\left[\frac{\%}{100\;\mathrm{mmHg}}\right]$$

where *P*_1_ is the lowest internal pressure, *P*_2_ is the highest internal pressure, *D*_1_ is the diameter at the pressure *P*_1_ and the *D*_2_ is the diameter at the pressure *P*_2_. The compliance of each graft was calculated from the mean of the compliance values of 10 cycles, and three grafts were tested per condition.

For the long‐term compliance assessment, grafts were tested over a period exceeding 5 h. The compliance measurements were conducted at 20 min intervals, with each measurement lasting 15 s. To calculate the mean compliance at each timepoint for each graft, data from the initial 10 pulse cycles within each interval was utilized, and three grafts were evaluated for each condition.

The same setup was also used to measure the pressurized inner diameter of the prothesis. For that purpose, the mean pressure of the pulse free flow was adjusted to 120 mmHg and the resulting outer diameter was measured using the laser micrometer. The pressurized inner diameter was then calculated using the following formula

(3) $$d_{\mathrm{pi}}=d_{\mathrm{pa}}-2\;t_{w}\;\left[\mathrm{mm}\right]$$

where *d*_pa_ is the pressurized outer diameter and *t*_w_ is the average wall thickness of the graft. The wall thickness was determined according to the method described above.

#### 2.4.4 Burst Strength

Burst strength values were measured in a custom‐made burst strength chamber equipped with a pressure sensor (Jumo Midas pressure transmitter; JUMO GmbH & Co. KG, Fulda, Germany) and an NE‐1000 SyringeONE Programmable Syringe Pump (New Era Pump Systems, Inc; Farmingdale, NY, USA). For each condition, rehydrated samples (*n* = 3) with areas of 1 cm^2^ were placed in the burst chamber, which was immersed in PBS buffer solution at 37 °C for 10 min and then exposed to increasing pressure by pumping PBS at a constant rate of 20 mL min^−1^ until the structural failure of the sample was detected as a sudden drop in the pressure recorded by LabVIEW (National Instruments). The highest pressure measured before failure was defined as the burst strength value. The pressure was recorded in mmHg. The accuracy of the pressure measurement was validated prior to testing using an analog blood pressure monitor.

For validation of the indirect burst strength tests, the burst strength of tubular samples was also evaluated, in accordance with the ISO requirements. The samples (length = 20 mm) were mounted on the burst chamber on one side and completely filled with PBS (Gibco, pH: 7.4, Life Technologies, Carlsbad, CA, USA) before being closed using a stopper and then immersed in the PBS buffer at 37 °C. The same parameters as in the indirect test were applied.

### 2.5 Cell Attachment

Vascular grafts under sterile conditions were produced and then cut using a 6 mm biopsy punch to produce samples fitting into a 96 well plate, with a surface area 0.32 cm^2^. All samples were rehydrated and washed in PBS for 3 h before seeding with human endothelial cells (HUVECs) isolated from human umbilical cords as previously described.^[^ ^43^ ^]^ Briefly, human umbilical cords were obtained after written informed consent at University Hospital Aachen (Aachen, Germany) and were provided through the RWTH Aachen University Centralized Biomaterial Bank (cBMB), in compliance with its regulations, following RWTH Aachen University Medical Faculty Ethics Committee approval (cBMB project number 323). HUVECs were cultured in endothelial growth medium 2 (EGM2, PromoCell, Heidelberg, Germany) supplemented with fetal calf serum, epidermal growth factor, basic fibroblast growth factor, insulin‐like growth factor, vascular endothelial growth factor 165, ascorbic acid, heparin, and hydrocortisone. The cells were expanded under standard culture conditions in a humidified 5% CO_2_ atmosphere at 37 °C. Only primary cells up to passage 4 were used for all the experiments. After the HUVECs reached 70–80% confluency, they were trypsinized, resuspended in 2 mL of EGM2 medium and counted. The cell suspension was then mixed with additional EGM2 media to achieve a cell concentration of 3 × 10^5^ cells mL^−1^. The scaffolds were placed inside a 96 well plate, and then 100 µL of cell suspension was pipetted onto the samples. The well plate was gently tilted in a circular fashion, to allow for the dispersion of the cells before being placed in an incubator (37 °C, 5% CO_2_) for 2 h to facilitate cell attachment to the scaffold. After incubation, the samples were washed three times with PBS before fixation with methanol‐free paraformaldehyde 4% (v/v) (Carl Roth, Karlsruhe, Germany) for 1 h at room temperature and again washed three times with PBS.

For actin filament visualization, the cells were permeabilized by incubation for 5 min in a Triton‐X 100 (0.1% (v/v)) in PBS solution and subsequently rinsed three times in PBS. They were then stained with phalloidin‐iFluor 488 conjugate (CaymanChemicals, Ann Arbor, MI, USA) at a dilution of 1:1000 in a bovine serum albumin (1% (v/v)) in PBS solution for 90 min at room temperature. After being rinsed three times with PBS, the DRAQ5 (1 µg mL^−1^) (Thermo Fisher Scientific, Waltham, MA, USA) in PBS solution was added onto the samples to stain the nuclei.

### 2.6 Statistical Analysis

Statistical differences were assessed either using a one‐way or two‐way analysis of variance (ANOVA) followed by the post hoc Holm–Šidák method. All experiments were conducted at least in triplicate (*n* ≥ 3). The results are presented as means ± SD. The threshold for statistical significance was *p* < 0.05 (*p < 0.05; **p < 0.01, ***p < 0.001, ****p < 0.0001). Any value of *p* > 0.05 was defined as nonsignificant. The statistical analysis was done using GraphPad Prism.

## 3 Results

### 3.1 Vascular Graft Production

**Figure 2:**
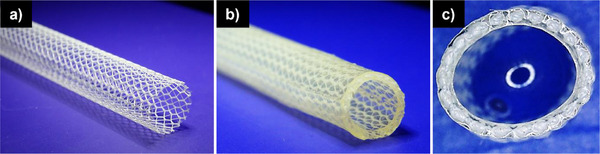
Textile‐reinforced vascular graft. a) Image of PET textile used for the reinforcement. b) Semilateral view of finished graft after embedding the PET textile mesh inside the ELR hydrogel via injection molding. c) Zoom‐in of the cross‐section of a graft to illustrate the coaxial position of the textile.

We successfully manufactured grafts composed of protein‐engineered elastin‐like matrix reinforced with a PET wrap‐knitted mesh (**Figure** **2**a). The grafts had an inner diameter of 4.86 ± 0.26 mm (±5.6% of mean value), a wall thickness of 689 ± 33 µm (±4.8% of mean value), and a length of at least 10 cm. Our new biohybrid vascular graft (Figure 2b) features a reduction of the wall thickness by 60% with respect to our previous design,^[^ ^20^ ^]^ leading to a wall thickness that is comparable to that of native blood vessels with similar diameters.^[^ ^44^, ^45^ ^]^ Assessment of the textile coaxiality along the graft, showed that the production method also guarantees a centered position of the textile reinforcement within the hydrogel wall matrix with only a ±3% variation (Figure 2c).

### 3.2 Effect of Drying Scheme on Graft Morphology and Structure

**Figure 3:**
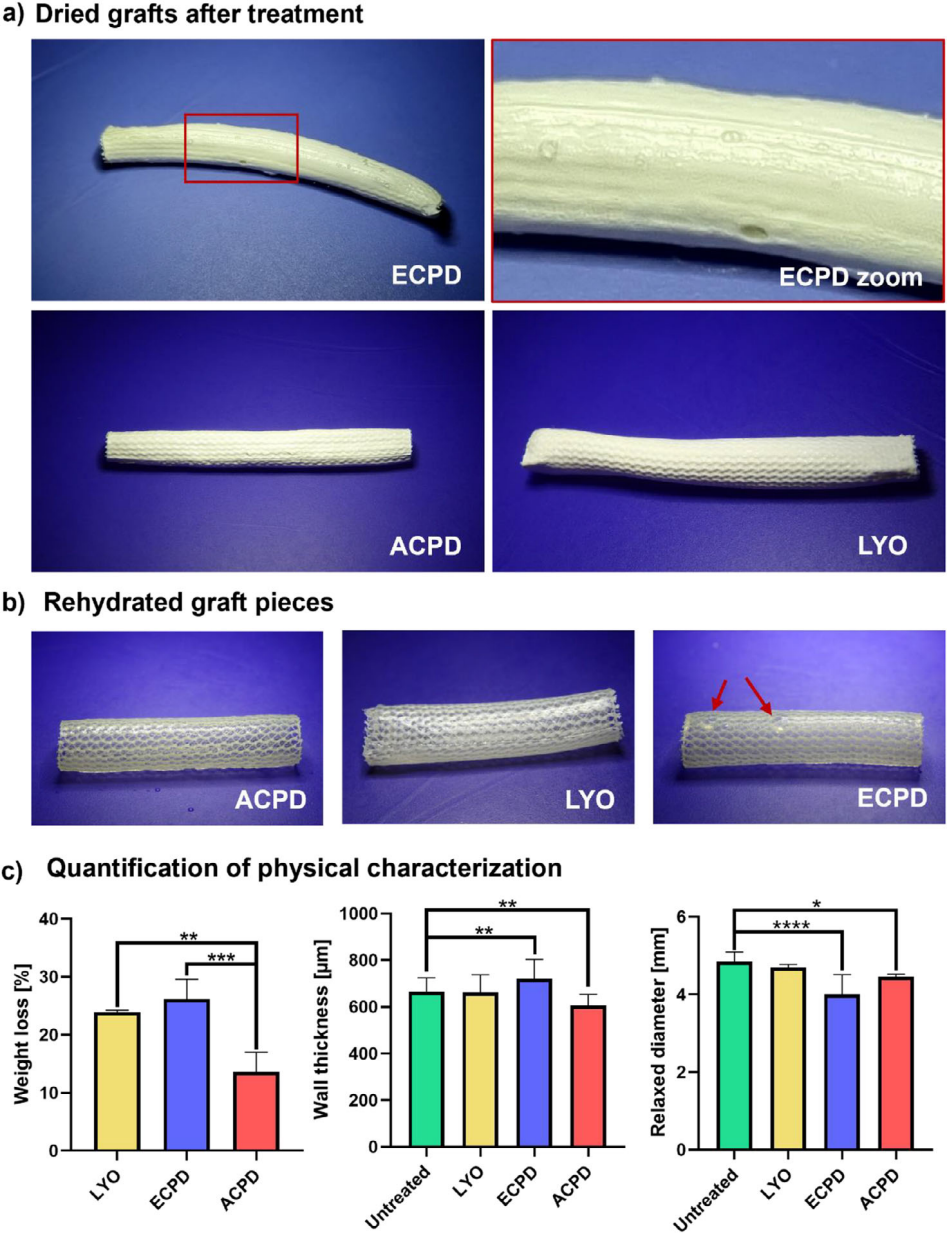
Physical characterization of the graft after drying. a) Representative images of dried grafts after the different treatments. The applied treatments were lyophilization (LYO), critical‐point drying after dehydration with ethanol (ECPD), and critical‐point drying after dehydration with acetone (ACPD). b) Representative images of rehydrated grafts, with the red arrows indicating bubble formation in the ECPD sample. c) Morphological evaluation of the rehydrated grafts (inner diameter, wall thickness and weight changes due to swellability of the ELR matrix) after submersion in PBS at 37 °C for 10 min. For the weight, the results are expressed as changes in percentage with respect to the control (untreated state). For wall thickness and inner diameter, the treatment groups were compared to the control (untreated). Sample number for all groups *n* = 3. Statistical differences were assessed using a one‐way ANOVA followed by post hoc Holm–Šidák method. The threshold for statistical significance was *p* < 0.05 (*p < 0.05; **p < 0.01, ***p < 0.001, ****p < 0.0001). Any value of *p* > 0.05 was defined as nonsignificant.

Macroscopic inspection of the grafts dried with ECPD revealed visible defects (**Figure** **3**a), while samples treated with LYO and ACPD showed no defects and generally possessed a more homogeneous appearance (Figure 3a). After rehydration, there was no macroscopic difference between the Untreated, LYO, and ACPD grafts, while the ECPD‐treated grafts exhibited bubble formation (Figure 3b). The graft weight after rehydration differed depending on the treatment (Figure 3c). Specifically, rehydrated grafts subjected to LYO and ECPD, experienced a weight loss of 23.9% ± 0.34% and 27.8% ± 2.97%, respectively, when compared to their nontreated counterparts. Notably, those treated with ACPD showed a significantly less pronounced decrease in swellability, with a weight loss of 13.6% ± 2.75%.

Regarding the impact of the drying scheme on the dimensions, the grafts treated with ECPD experienced an increase of the wall thickness of 8.50% ± 12.3%, while the wall thickness of the ACPD group decreased by 8.58% ± 7.03% (Figure 3c). The LYO group showed no significant changes in either wall thickness or inner diameter. Both CPD treatments had a significant effect on the dimensions of the relaxed inner diameter. Specifically, the diameter of rehydrated grafts decreased by 17.26% ± 3.82% after ECPD and 7.84% ± 1.11% after ACPD drying, respectively (Figure 3c).

**Figure 4:**
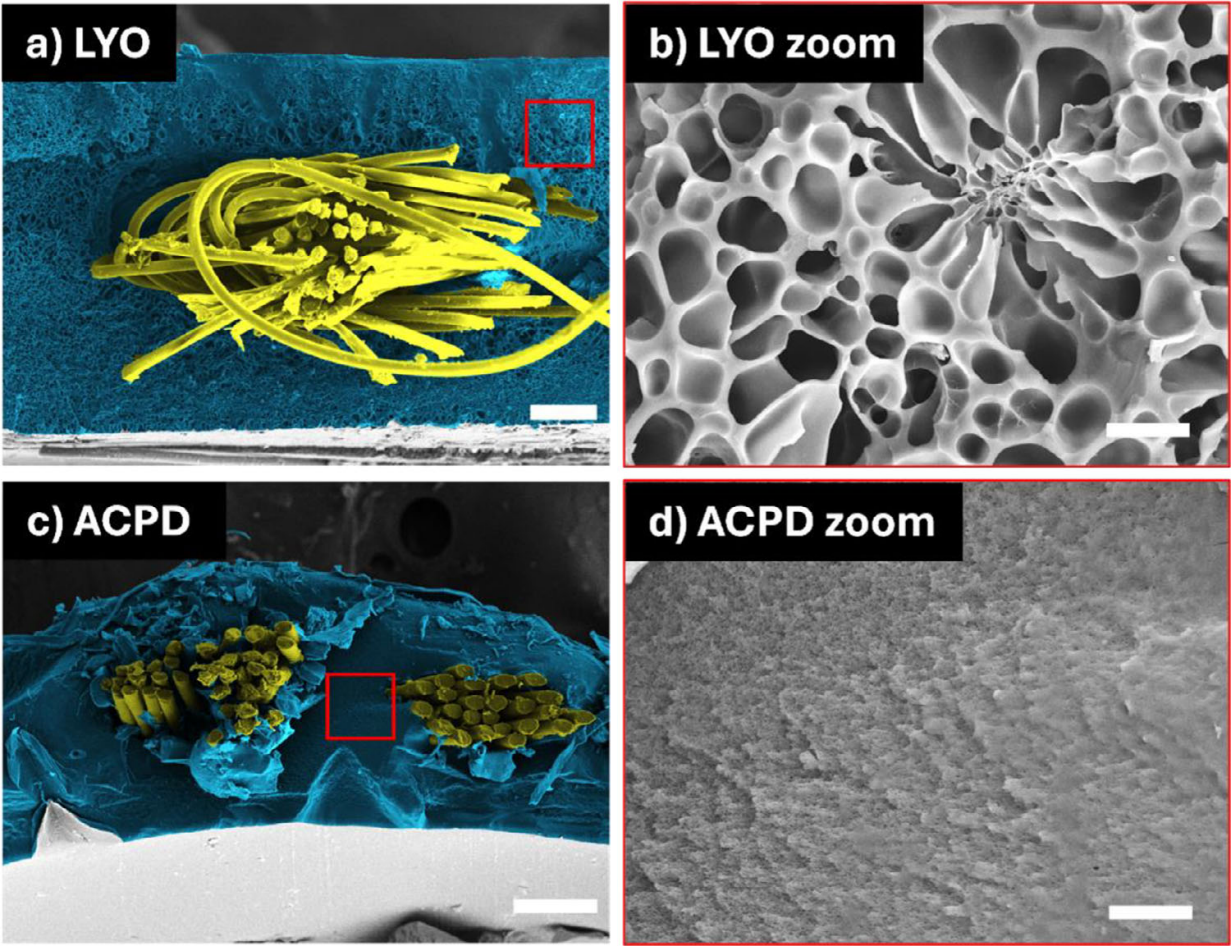
Representative SEM pictures of LYO and CPD dried grafts in dry state. a) Cross‐section of LYO dried graft, with the PET textile colored in yellow and the cutting surface of the ELR matrix colored in blue with the software MountainsSEM (Digital Surf, France). b) Magnified view of the microporous ELR matrix of a lyophilized graft. c) Cross‐section of ACPD dried graft, with the PET textile colored in yellow and the cutting surface of the ELR matrix colored in blue with the software MountainsSEM (Digital Surf, France). d) Magnified view of the nanoporous ELR matrix of an ACPD dried graft. Scale bars: (a,c): 100 µm; (b,d): 10 µm.

Analysis of the scaffolds by SEM showed that LYO led to the formation of micropores (**Figure** **4**a,b), with an average pore size of 5.1 ± 2.2 µm. This microporosity was absent in the CPD samples, which showed a very tight matrix with sub‐micrometer pores (Figure 4c,d). The observed porosity in the lyophilized samples persisted after rehydration (Figure S1, Supporting Information). SEM visualization also confirmed the centered position of the textile inside the ELR matrix (Figure 4a,c).

### 3.3 Mechanical Characterization

The grafts were subjected to a series of mechanical tests, following the protocols outlined in ISO 7198.^[^ ^40^ ^]^

#### 3.3.1 Tensile Testing: Impact of the Drying Method on Elastic Behavior

**Figure 5:**
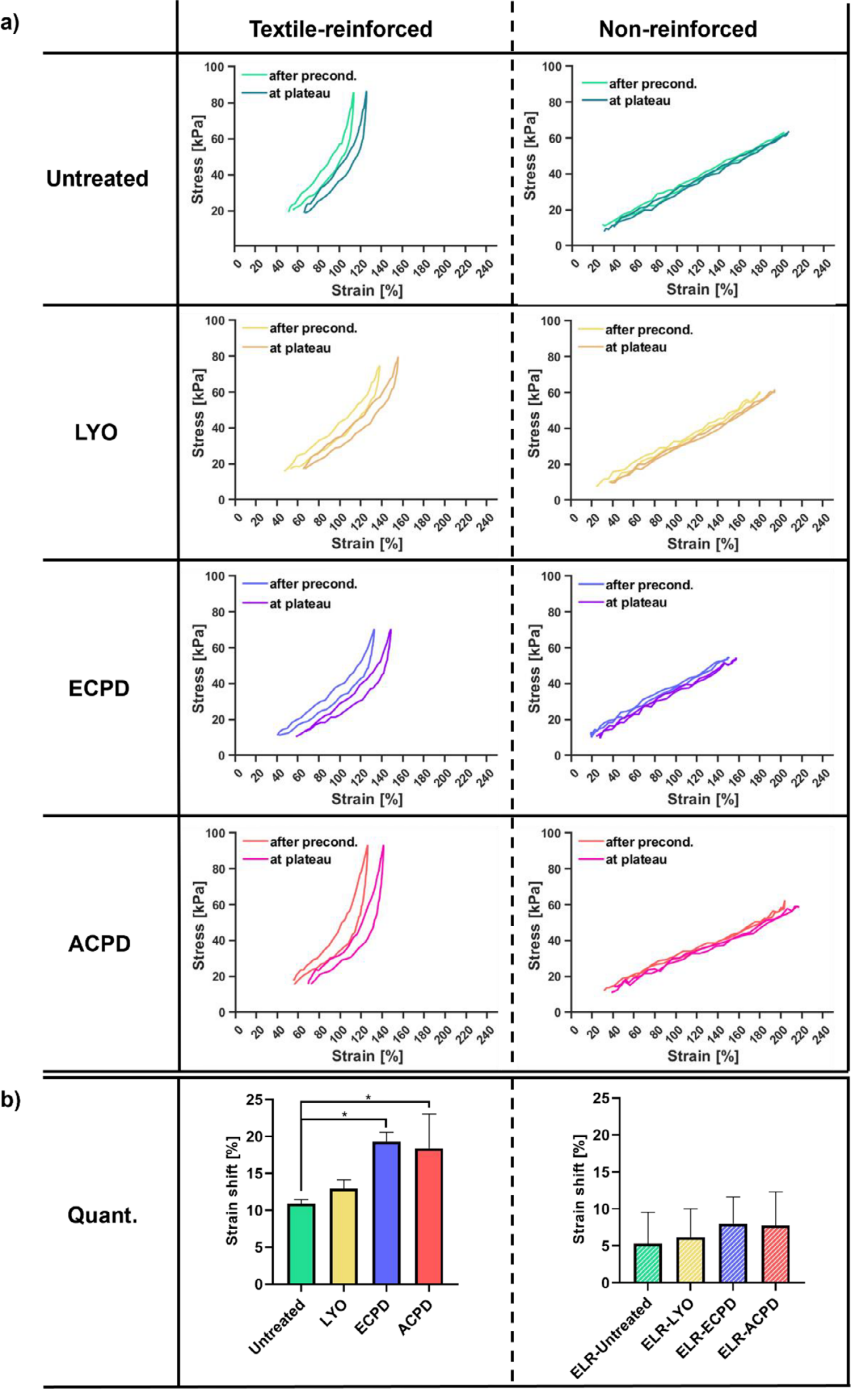
Strain‐shift from cycle 5 to cycle 150 during cyclic testing at 37 °C for different treatment groups. a) Stress–strain curves for textile‐reinforced and nonreinforced, untreated grafts as well as subjected to the different drying methods (LYO, ECPD, ACPD). b) Calculated strain‐shifts for textile‐reinforced (left panel) and nonreinforced (right panel), untreated grafts as well as subjected to different drying methods (LYO, ECPD, ACPD). Depicted as mean (*n* = 3) ± SD. Statistical differences were assessed using a one‐way ANOVA followed by post hoc Holm–Šidák method. The threshold for statistical significance was *p* < 0.05 (*p < 0.05). Any value of *p* > 0.05 was defined as nonsignificant.

We conducted cyclic tensile tests with ring samples (up to 200 cycles), in accordance with ISO 7198,^[^ ^40^ ^]^ to investigate if plastic deformation of the graft takes place, and to determine the influence of the treatment on the Young's modulus. An initial strain‐shift was observed for all reinforced grafts up to cycle 150 (**Figure** **5**a, left panel), after which all sample groups reached a stable point, and no further change occurred. Such strain‐shift was more pronounced for ECPD (19.31% ± 1.03%) and ACPD (18.38% ± 3.80%), while no significant differences were found between the Untreated group (10.91% ± 0.44%) and LYO (12.93% ± 0.98%) (Figure 5b, left panel).

The nonreinforced ELR samples, independent of treatment, exhibited a less pronounced strain‐shift (Figure 5a, right panel) with no statistical difference between the different groups (Figure 5b, right panel). The strain‐shifts were 5.32% ± 3.46% (Untreated), 6.15% ± 3.15% (LYO), 8.00% ± 2.94% (ECPD), and 7.79% ± 3.69% (ACPD) (Figure 5a,b, right panel).

**Figure 6:**
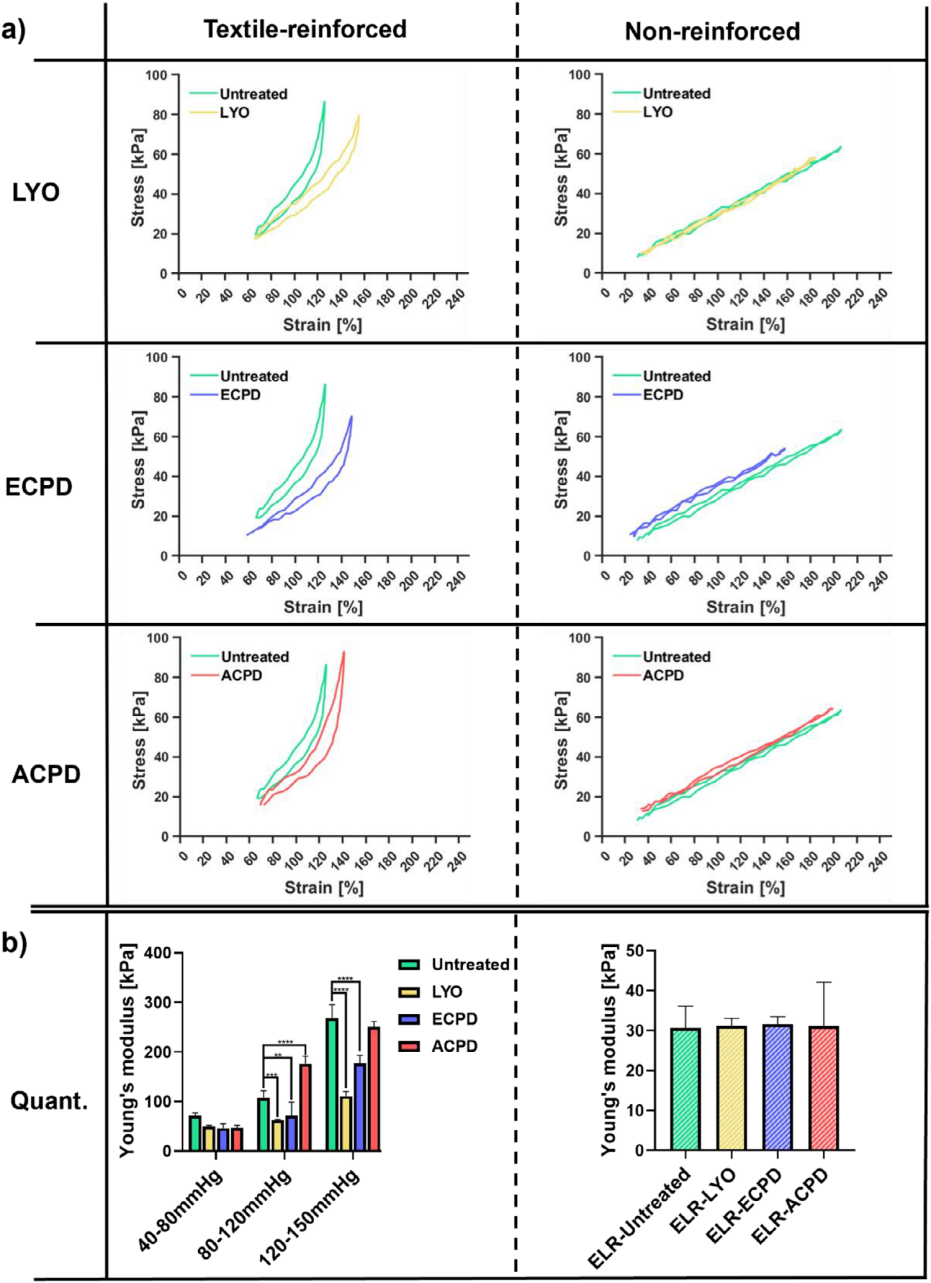
Comparison of stress–strain behavior of textile reinforced and nonreinforced grafts at 37 °C, upon different treatments. The curves were recorded upon reaching a plateau, at cycle 150. a) Mean (*n* = 3). Stress–strain curves of differently treated grafts (LYO, ECPD, ACPD) compared to untreated ones. b) Calculated Young's moduli for the pressure ranges 40/80, 80/120, and 120/150 mmHg for the textile‐reinforced graft, as well as the calculated Young's moduli for the nonreinforced grafts, depicted as mean (*n* = 3) ± SD. Statistical differences were assessed using either a one‐way (b, right panel) or two‐way (b, left panel) ANOVA followed by post hoc Holm–Šidák method. The threshold for statistical significance was *p* < 0.05 (*p < 0.05). Any value of *p* > 0.05 was defined as nonsignificant.

The reinforced grafts exhibited nonlinear strain–stress relation within the tested range (**Figure** **6**a, left panel), meaning that they exhibited non‐Hookean behavior. In contrast, the nonreinforced samples, made of solely ELR, showed linear behavior regardless of the treatment (Figure 6a, right panel). Due to the non‐Hookean behavior of the reinforced grafts, we calculated the Young's modulus for three different force ranges, representing the pressure ranges 40–80 mmHg, 80–120 mmHg, and 120–150 mmHg to gain comprehensive data about the graft behavior (Figure 6b, left panel). The nonreinforced samples showed Hookean behavior within the applied force range, so segmentation was not applied for these groups (Figure 6b, right panel).

None of the treatments led to a statistically significant change in Young's modulus for the 40–80 mmHg range. Specifically, the Young's moduli were 71.72 ± 4.13 kPa, 50.24 ± 1.35 kPa, 45.88 ± 7.24 kPa, and 46.37 ± 4.69 kPa for the Untreated, LYO, ECPD, and ACPD groups, respectively. However, a tendency toward a higher Young's modulus for the untreated group is already visible and becomes statistically significant at higher pressure ranges (Figure 6b, left panel). Specifically, for the 80–120 mmHg range, both LYO as well as ECPD showed a significantly lower Young's modulus (61.40 ± 2.08 kPa and 71.79 ± 22.11 kPa, respectively) than the untreated counterparts (106.9 ± 11.81 kPa). On the other hand, ACPD led to an increase of the Young's modulus to 175.84 ± 11.81 kPa. For the 120–150 mmHg, ACPD is the only treatment that did not result in a change in Young's modulus when compared to the Untreated group, with the values being 250 ± 8.95 kPa and 268.23 ± 21.95 kPa respectively. LYO and ECPD both led to a decrease of the Young's moduli, with values of 110.91 ± 7.82 kPa and 177.18 ± 12.88 kPa, respectively (Figure 6b, left panel).

The nonreinforced samples showed no statistical difference for any of the applied treatments, with the Young's moduli of 30.69 ± 4.42 kPa, 31.28 ± 1.47 kPa, 31.60 ± 1.53 kPa, and 31.19 ± 8.92 kPa for the Untreated, LYO, ACPD, and ECPD groups, respectively (Figure 6b, right panel).

#### 3.3.2 Breaking Stress

**Figure 7:**
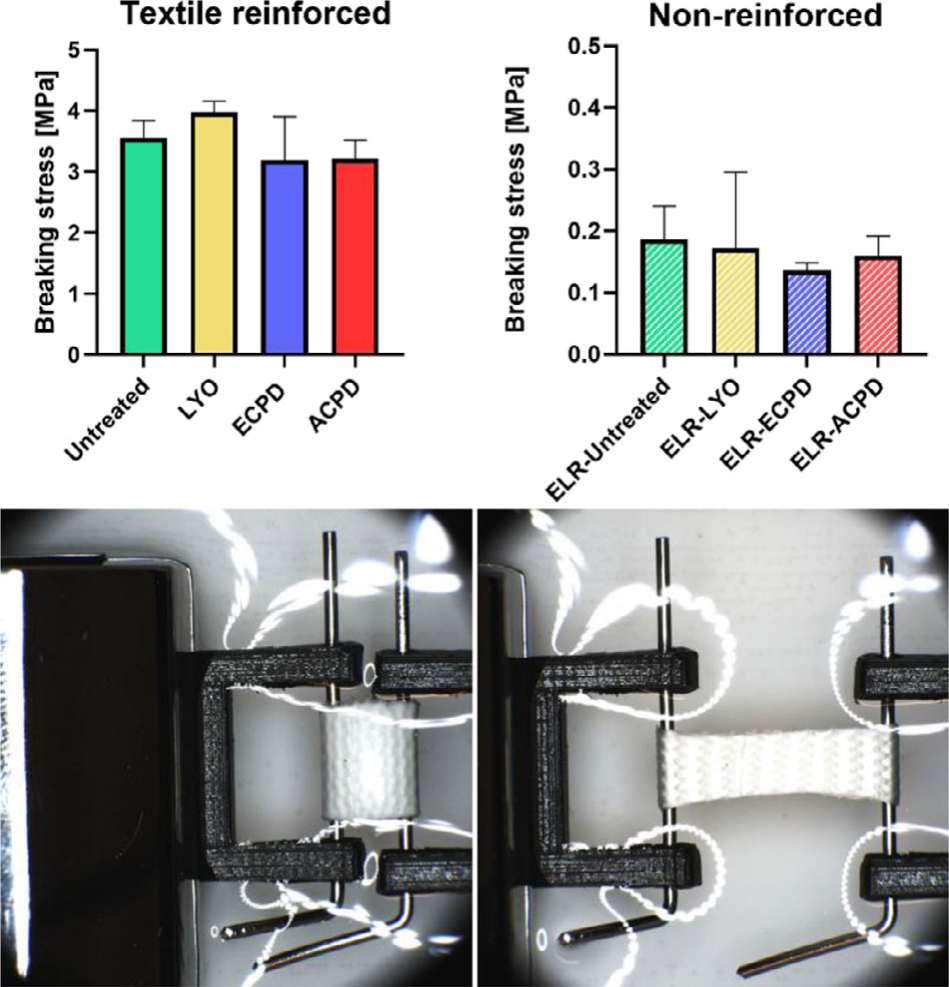
Maximum break stress for textile‐reinforced and nonreinforced samples, untreated and treated (LYO, ECPD, ACPD). Images show samples submerged in PBS at 37 °C and mounted on pins, relaxed (left) and stretched before break (right). Values depicted as mean (*n* = 3) ± SD. Statistical differences were assessed using a one‐way ANOVA followed by post hoc Holm–Šidák method. The threshold for statistical significance was *p* < 0.05 (*p < 0.05). Any value of *p* > 0.05 was defined as nonsignificant.

No significant changes in breaking stress were observed for the different treatment groups (**Figure** **7**). Specifically, the breaking stress for the textile reinforced samples was 3.6 ± 0.2 MPa, 4.0 ± 0.1 MPa, 3.2 ± 0.6 MPa, and 3.2 ± 0.2 MPa for the Control, LYO, ECPD, and ACPD groups, respectively (Figure 7, left panel)

The nonreinforced ELRs exhibit significantly lower break stresses than their textile‐reinforced counterparts, with values of 0.19 ± 0.04 MPa (Untreated), 0.17 ± 0.1 MPa (LYO), 0.14 ± 0.01 MPa (ECPD), and 0.16 ± 0.03 MPa (ACPD), respectively (Figure 7, right panel).

#### 3.3.3 Suture Retention

**Figure 8:**
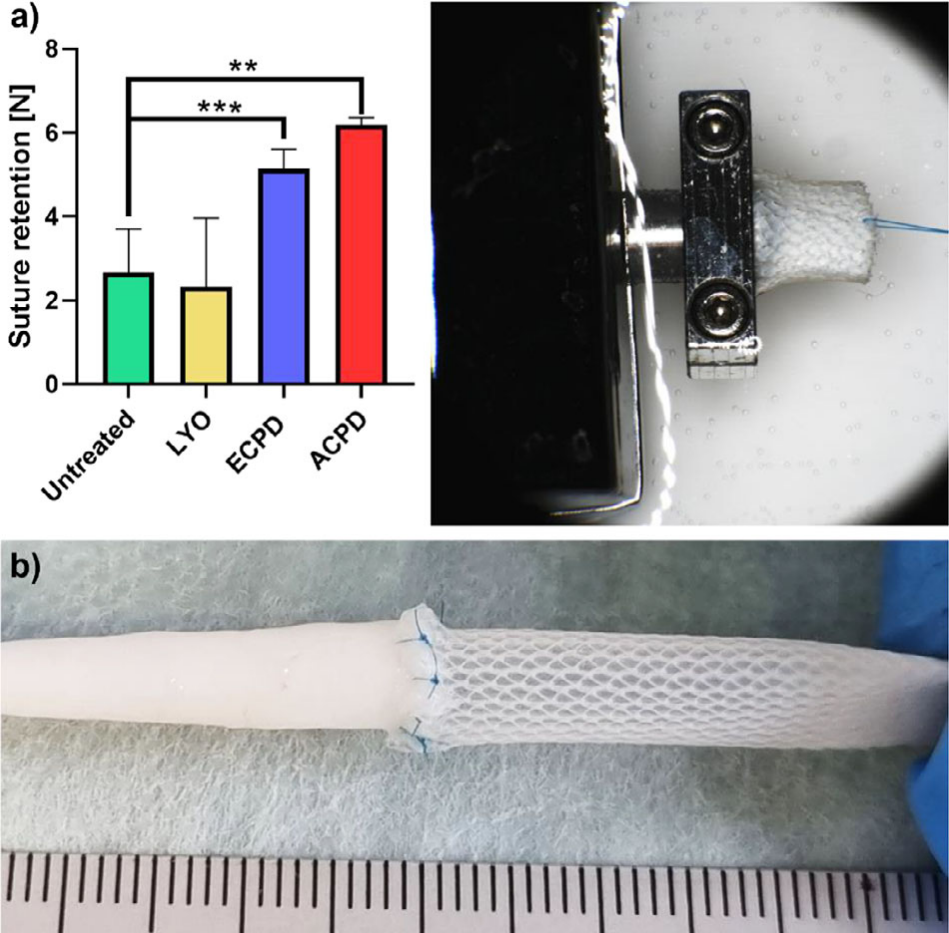
a) Suture retention strength with 6‐0 prolene suture at 37 °C of untreated and treated grafts (LYO, ECPD, ACPD). The image on the left shows a mounted graft sample submerged in PBS with a blue suture being pulled by the tensile tester. Values depicted as mean (*n* = 4) ± SD. b) Anastomosis between biohybrid graft and porcine carotid artery. Ruler resolution is 1 mm. Statistical differences were assessed using a one‐way ANOVA followed by post hoc Holm–Šidák method. The threshold for statistical significance was *p* < 0.05 (*p < 0.05; **p < 0.01, ***p < 0.001). Any value of *p* > 0.05 was defined as nonsignificant.

Another essential characteristic that a vascular graft has to fulfill is sufficient suture retention, to ensure that the prothesis stays attached to the native vasculature (anastomosis) without leakage or rupture. The applied drying method had a significant effect on the suture retention outcomes (**Figure** **8**a), but importantly, all grafts showed values comparable to or higher than native vessels. Specifically, the suture retention force was 2.66 ± 0.9 N and 2.33 ± 1.4 N for the Untreated and LYO grafts, respectively, while it significantly increased to 5.15 ± 0.4 N and 6.2 ± 0.1 N for ECPD and ACPD. Therefore, the suture retention of the ECPD and ACPD treated grafts was 193% and 233% higher, respectively, than that of the control group.

We also performed an anastomosis between our grafts and a native porcine carotid artery (Figure 8b), followed by perfusion and pulling tests (Figure S2 and Video S1, Supporting Information), to further verify the suturability of rehydrated LYO and ACPD grafts. These tests showed no leakage under a flow of 100 mL min^−1^ at the anastomosis site and the suture stayed intact even when applying a 10 N pulling force.

#### 3.3.4 Compliance Behavior

**Figure 9:**
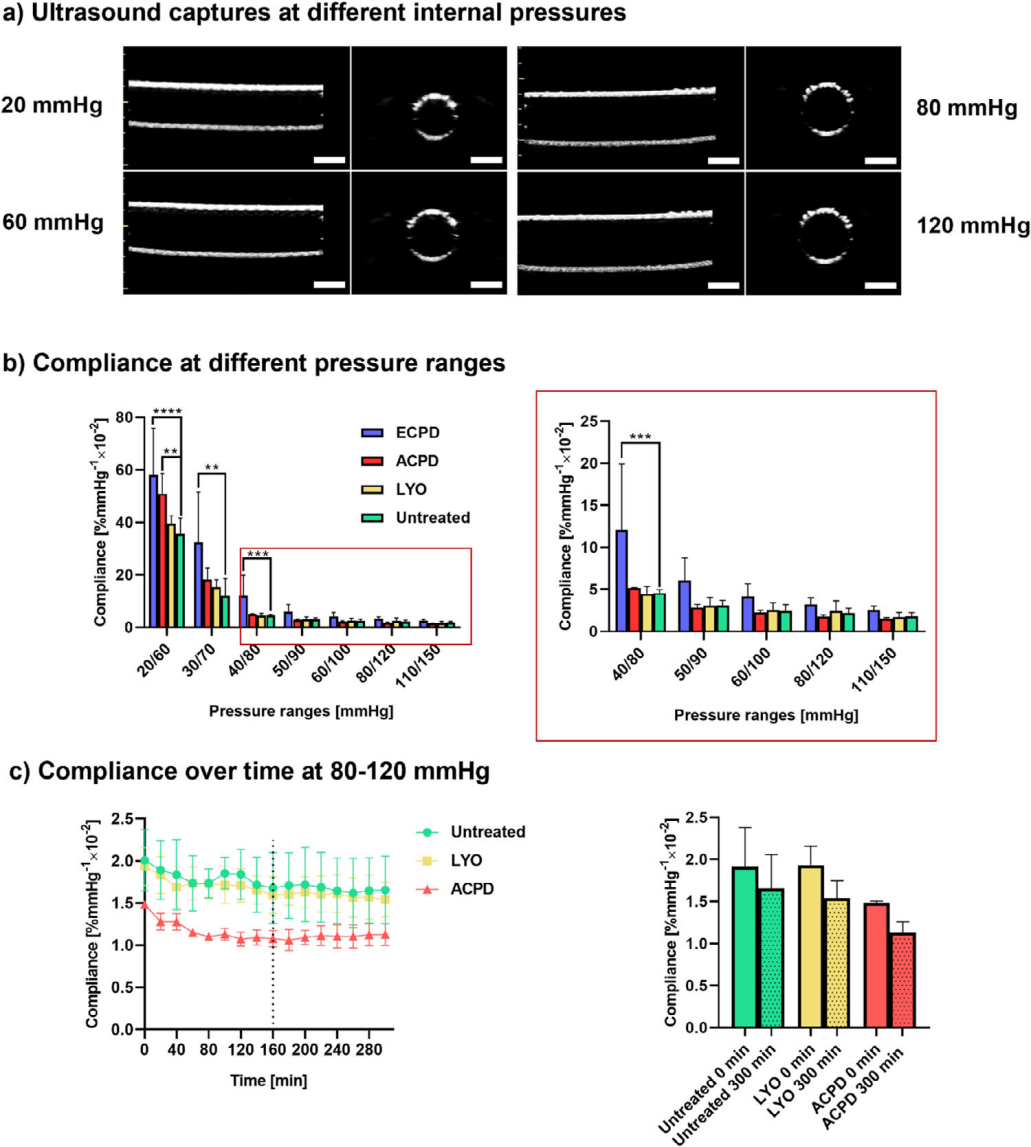
Compliance of treated (dried and rehydrated) and untreated (control) grafts at 37 °C. The drying treatments were LYO, ECPD, and ACPD. a) Representative ultrasound captures at 20, 60, 80, and 120 mmHg. b) Compliance behavior from low (20/60 mmHg) to high (110/150 mmHg) pressure and in right panel a magnification of the 40–150 mmHg range. c) Compliance behavior at 80–120 mmHg over 5 h for Untreated, LYO, and ACPD groups. Dotted line indicates the pressure after which no further decrease is observed (plateau). Scale bars (a): 5 mm. Values depicted as mean (*n* = 3) ± SD. Statistical differences were assessed using either a one‐way (c) or two‐way (b) ANOVA followed by post hoc Holm–Šidák method. The threshold for statistical significance was *p* < 0.05 (*p < 0.05; **p < 0.01, ***p < 0.001, ****p < 0.0001). Any value of *p* > 0.05 was defined as nonsignificant.

The expansion–recoiling behavior of the graft under physiological‐like pulsatile pressure was initially monitored by ultrasound imaging (**Figure** **9**a; Video S2, Supporting Information), which enables to visualize not only the abluminal but also the intraluminal dimensional changes, as well as the effect of the applied pressure on the wall thickness. The wall thickness stayed constant regardless of the pressure applied, and therefore, the change in outer diameter can be directly translated to a change in inner diameter.

The compliance of untreated grafts was 12.1% ± 5.3% mmHg^−1^ × 10^−2^, 4.6% ± 0.3% mmHg^−1^ × 10^−2^, 3.1% ± 0.5% mmHg^−1^ × 10^−2^, 2.2% ± 0.5% mmHg^−1^ × 10^−2^, and 1.8% ± 0.3% mmHg^−1^ × 10^−2^ for the 30/70 mmHg, 40/80 mmHg, 50/90 mmHg, 80/120 mmHg, and 110/150 mmHg pressure range, respectively (Figure 9b).

The LYO grafts exhibited 15.3% ± 2.4% mmHg^−1^ × 10^−2^, 4.5% ± 0.7% mmHg^−1^ × 10^−2^, 3.1% ± 0.5% mmHg^−1^ × 10^−2^, 2.4% ± 1.0% mmHg^−1^ × 10^−2^, and 1.7% ± 0.5% mmHg^−1^ × 10^−2^ of compliance for 30/70, 40/80, 50/90, 80/120, and 110/150, respectively, matching that of the untreated group.

The ECPD grafts exhibited a significant increase in compliance for lower pressure ranges (20/60, 30/70, and 40/80) with 58% ± 14.52% mmHg^−1^ × 10^−2^, 32.39% ± 15.61% mmHg^−1^ × 10^−2^, and 12.1% ± 6.4% mmHg^−1^ × 10^−2^, respectively. This treatment group also showed higher variability than the other groups, speaking for a less reproducible drying option of our graft concept. The grafts showed compliance values of 6.03% ± 2.2% mmHg^−1^ × 10^−2^ (for 50/90 mmHg), 3.2% ± 0.6% mmHg^−1^ × 10^−2^ (for 80/120 mmHg) and 2.6% ± 0.4% mmHg^−1^ × 10^−2^ (for 110/150 mmHg). The ACPD‐treated grafts also showed an increase in compliance compared to the control for the lowest pressure range tested (20/60 mmHg), with a compliance of 50.9% ± 6% mmHg^−1^ × 10^−2^. However, the ACPD grafts showed the same compliance behavior as the Untreated group for all higher pressure ranges. Specifically, the compliance values were 18.12% ± 3.71% mmHg^−1^ × 10^−2^ (30/70 mmHg), 5.13% ± 0.05% mmHg^−1^ × 10^−2^ (40/80 mmHg), 2.87% ± 0.29% mmHg^−1^ × 10^−2^ (50/90 mmHg), 2.26% ± 0.23% mmHg^−1^ × 10^−2^ (60/100 mmHg), 1.78% ± 0.15% mmHg^−1^ × 10^−2^ (80/120 mmHg), and 1.48% ± 0.14% mmHg^−1^ × 10^−2^ (110/150 mmHg).

We also investigated the compliance behavior over a prolonged period of time, in order to evaluate if the stretching–recoiling capability remains stable over time, or if there were signs of dilation/aneurysm (Figure 9c). A slight reduction in compliance was observed at early timepoints for all investigated groups. However, this trend was not statistically significant, and a plateau was reached at 160 min, which corresponds to ≈10 000 pulse cycles.

#### 3.3.5 Burst Pressure and Pressurized Diameter

**Figure 10:**
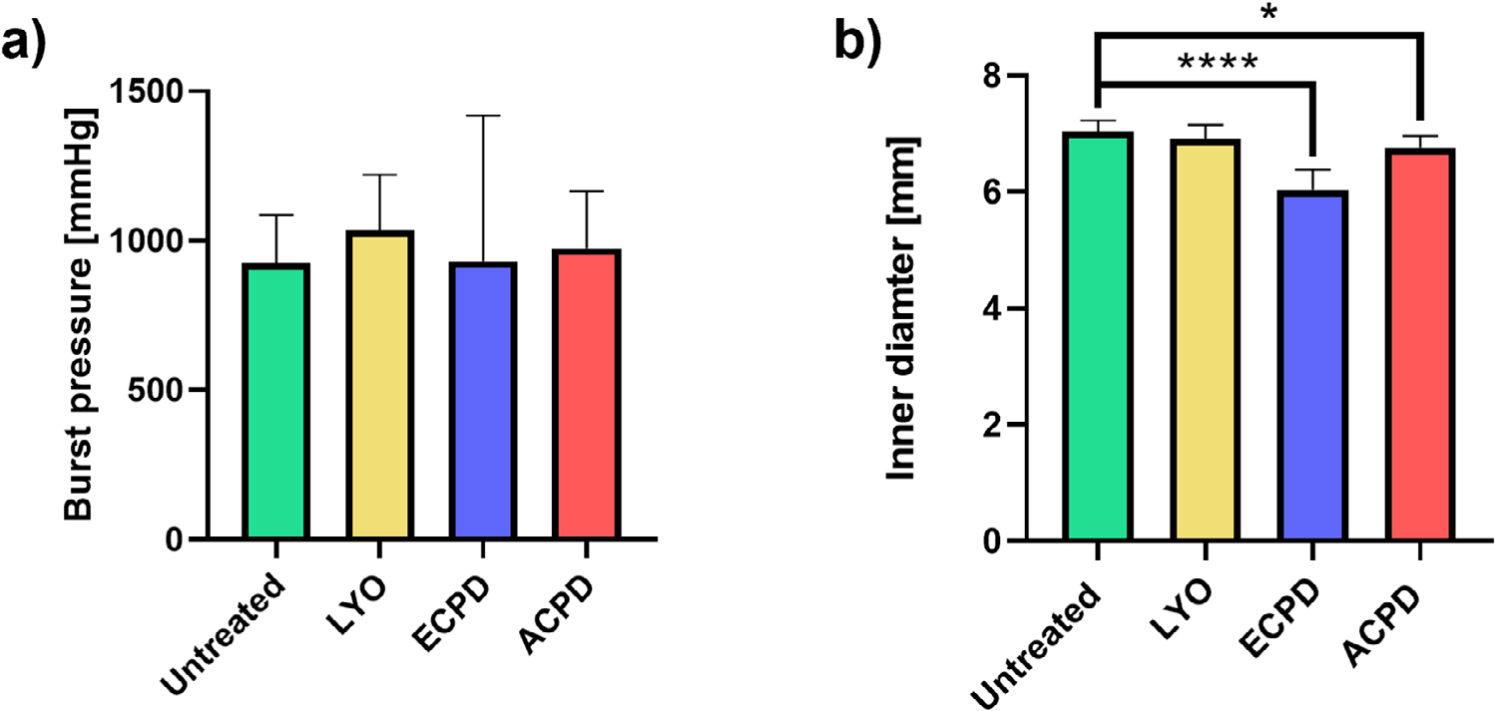
a) Burst pressure for the untreated grafts as well as different treatment groups (LYO, ECPD, ACPD) in PBS at 37 °C. b) Inner diameter of all treatment groups (Untreated, LYO, ECPD, ACPD) at a pressure of 120 mmHg. Values depicted as mean (*n* = 3) ± SD. Statistical differences were assessed using a one‐way ANOVA followed by post hoc Holm–Šidák method. The threshold for statistical significance was *p* < 0.05 (*p < 0.05; **p < 0.01, ***p < 0.001, ****p < 0.0001). Any value of *p* > 0.05 was defined as nonsignificant.

Besides compliance and suture retention, burst pressure is one of the dominating factors when considering graft performance. None of the applied drying methods significantly affected the average burst pressure of the vascular graft (**Figure** **10**a). The vascular grafts exhibited a burst strength of 924 ± 152 mmHg, 1034 ± 179 mmHg, 930 ± 467 mmHg, and 973 ± 184 mmHg for Untreated, LYO, ECPD, and ACPD, respectively.

Another important characteristic of vascular grafts is their pressurized inner diameter, to ensure that their diameter matches that of the blood vessel at the intended implantation site. Intraluminal pressurization of the vascular grafts resulted in an increase of the inner diameter for all groups. The degree of this expansion depended on the applied treatment. Compared to the Untreated group, ECPD showed the most pronounced decrease in pressurized inner diameter, while ACPD showed a slight decrease and LYO led to no change (Figure 10b). The Untreated grafts exhibited a pressurized inner diameter of 7.05 ± 0.18 mm while rehydrated ECPD and ACPD samples reached 6.04 ± 0.32 mm and 6.76 ± 0.19 mm, respectively. These results also demonstrate that the shrinkage observed in the relaxed diameter (Figure 3c) persists under internal pressure.

### 3.4 Cell Attachment

**Figure 11:**
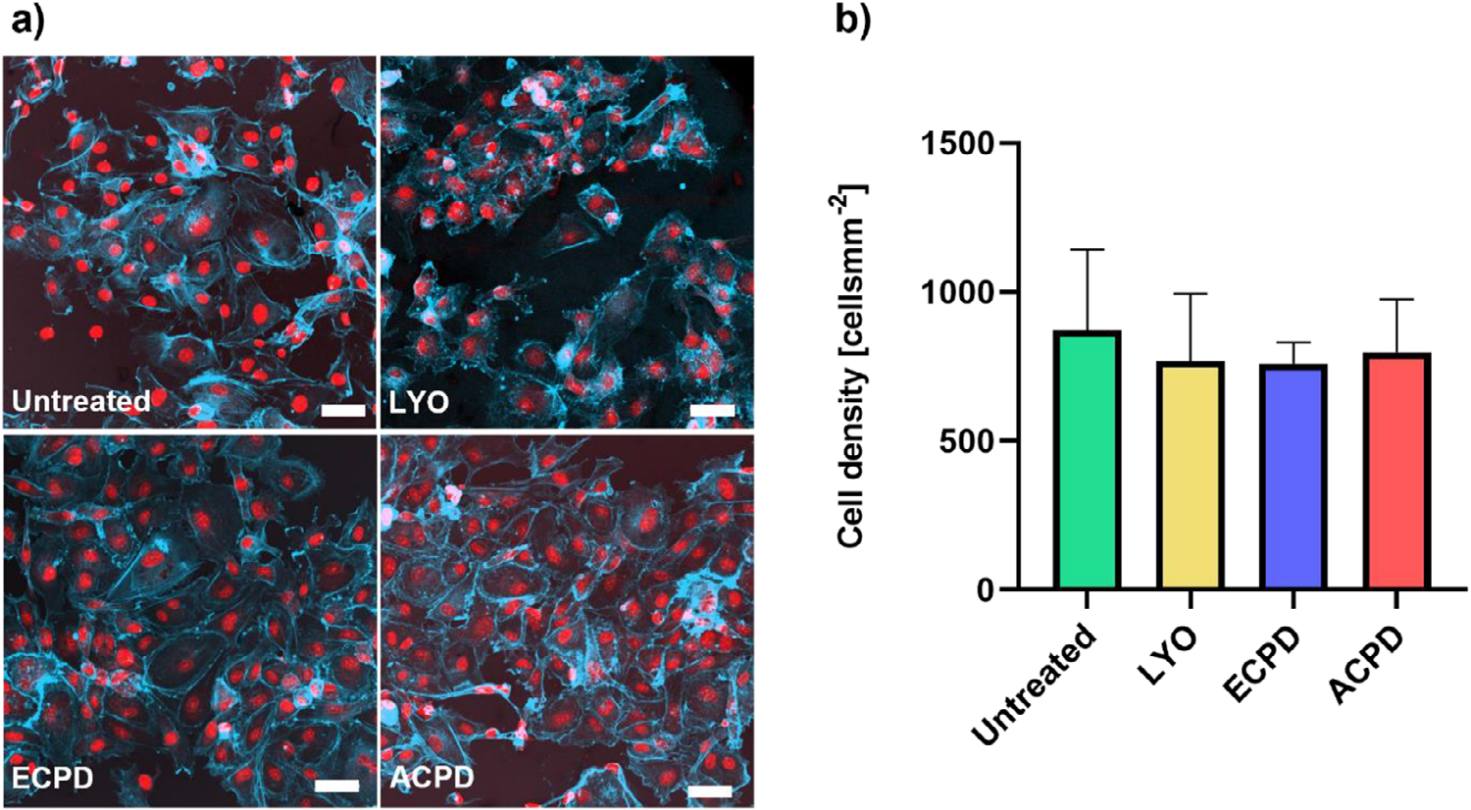
Impact of drying scheme on cellular attachment. a) Representative confocal images of graft samples, subjected to the different treatments and seeded with HUVECs. Nuclei are stained with DRAQ5 (red) and actin‐filaments with phalloidin (blue). b) Quantification of cell attachment for the different treatments. Values depicted as mean (*n* = 3) ± SD. Statistical differences were assessed using a one‐way ANOVA followed by post hoc Holm–Šidák method. The threshold for statistical significance was *p* < 0.05 (*p < 0.05). Any value of *p* > 0.05 was defined as nonsignificant. Scale bars: 10 µm.

To assess the potential impact of drying and subsequent rehydration on cellular adhesion, we investigated the attachment of primary HUVECs to graft segments subjected to various treatment protocols. The visualization via confocal microscopy showed no morphological changes and the semiquantitative analysis showed comparable density values for all groups (**Figure** **11**a,b). Therefore, none of the applied methods influenced the cell attachment.

## 4 Discussion

This study elucidates the optimized production of textile‐reinforced ELR‐based vascular grafts and how the characteristics of these grafts can be preserved or influenced through drying. The here presented biohybrid vascular graft (Figure 2) represents a refined version with regard to our previous one,^[^ ^20^ ^]^ by eliminating the macroporosity, as well as the e‐spun PCL layer. Thus, the new graft benefits from a straightforward design and production process to facilitate increased reproducibility. The precise positioning of the textile within the hydrogel matrix is one of the main challenges in the production of textile‐reinforced hydrogels. This issue is particularly critical when fabricating long, small‐diameter scaffolds, such as those intended for small diameter vascular prostheses. In these cases, it is essential that the textile is concentrically located within a hydrogel of physiologically relevant thickness, forming the vascular graft wall, without being exposed on any of the two surfaces. Even slight deviations in textile positioning can result in uneven mechanical load distribution, biointegration and, consequently, unpredictable long‐term performance. Though optimization of the production process, the resulting graft now combines the best of both the biological and the technical world: i) a bioinspired hydrogel, highly elastic and bioresorbable, for in situ remodeling,^[^ ^20^, ^22^, ^37^, ^46^, ^47^, ^48^ ^]^ and ii) a technical textile, for mechanical stability right after implantation, fabricated from a material that has proven its effectiveness and long‐term stability in decades of clinical use.^[^ ^49^ ^]^ We demonstrated in previous studies that ELR hydrogels, containing the proteolytic cleavage sequence Asp–Arg–Ile–Arg (DRIR), were degraded completely within 12 weeks when implanted subcutaneously in mice.^[^ ^36^ ^]^ Considering that the environmental conditions in a vascular graft, such as the blood flow, mechanical forces, and presence of various enzymes, are different from those in subcutaneous tissue, the remodeling time might differ for the presented prothesis. In vivo studies more closely replicating conditions of the intended medical application as vascular grafts would be necessary to establish a more exact timeframe. Importantly, the recombinant production of the ELRs enables to easily tune the number and the type of cleavage sites, allowing for the adjustment of the degradation rate, if needed.^[^ ^50^ ^]^

The characterization of the vascular grafts considered the practical aspects of implantation procedures. Specifically, rehydration at room temperature is preferred, as it minimizes the need for additional equipment, making the procedure more straightforward and accessible for medical professionals. Based on this consideration, all tests were conducted using prostheses that were rehydrated at room temperature. To ensure physiological relevance, the tests were then performed at 37 °C, replicating the conditions the prostheses would experience after implantation.

The morphological characterization of the graft after rehydration showed that the drying method can significantly influence the dimensional characteristics of the prosthesis. For example, ACPD leads to a decrease in both inner diameter and wall thickness, while ECPD treated grafts experienced an increased in wall thickness and a decrease in inner diameter when compared to the Untreated group (Figure 3c). The differences in the relaxed diameter between the ACPD and ECPD groups could therefore be attributed to the increased wall thickness in the ECPD samples, as this would result in a narrower lumen.

The morphological differences observed in the samples dried via CPD after either immersion in 100% ethanol or acetone clearly show the importance of making the right choice of intermediate fluid during the CPD process. Such differences could be due to the less collapsed state of the ELRs when immersed in ethanol as compared to their appearance in acetone (Figure S3, Supporting Information). The fast increase in pressure and the change in solvent, from ethanol to liquid CO_2_, apparently induces a rapid collapse of the matrix. Ethanol might not be able to fully diffuse out of the material and become entrapped, leading to local swollen areas in the material. When the entrapped ethanol is then replaced by CO_2_, these places could lead to bubble formation, explaining the observed inhomogeneity of the grafts. In the case of ACPD, a compaction of the matrix could already be observed when the grafts were placed in 100% acetone. It resulted in most of the liquid being expelled from the matrix, expressed by the grafts turning rigid during the exchange of PBS with acetone. This compaction persisted after rehydration, but importantly, they recovered their flexible and elastic nature. These morphological changes would also explain the weight loss that can be observed for both groups (Figure 3c). Previous studies have shown that CPD of hydrogel structures typically results in a more compact matrix structure and a concomitant decrease in water uptake.^[^ ^51^, ^52^ ^]^

Drying via LYO introduced porosity (Figure 4), which was persisting even after rehydration (Figure S1, Supporting Information). This is intriguing, considering that the network was already completely formed when the porogens (ice crystals) were introduced into the matrix. One potential explanation for this phenomenon is that the formation of ice crystals, even after the ELR matrix has been crosslinked, leads to the creation of microdomains. In these areas, the ELRs could become more compact as they are compressed by the ice‐crystal porogens. This higher compaction could eventually favor supramolecular interactions between the ELR molecules, as described for other hydrogels,^[^ ^53^, ^54^ ^]^ and accordingly, the ELRs do not reoccupy that space once the porogen is removed (ice crystal is sublimated). The process of soaking the grafts in PBS and then freeze‐drying them may also influence the formation of pores in the material. Specifically, the salt concentration in PBS may influence the ice crystal formation, by progressively slowing down the freezing rate along the interface of the ice crystals due to the phenomena called freeze concentration.^[^ ^55^ ^]^ This increases the time for ice crystals to grow. In addition, the crystallized salts of the PBS might act as porogens, which will be washed out upon rehydration.^[^ ^56^ ^]^ This change in structure would also persist even after the scaffold is rehydrated in PBS due to the tight interaction established with the surrounding ELR chains when the porogen was present. The tight interactions between the ELR chains would reduce the available binding surface for water and thereby reduce the water uptake capability of the scaffold after LYO, explaining the observed weight loss (Figure 3c). Usually, LYO is applied on non‐crosslinked hydrogel precursors to introduce porosity, as the network will then form after the ice crystals and thereby following their structure.^[^ ^34^ ^]^ This finding, can have implications for the use of LYO to increase the surface area and concomitantly the cell attachment and ingrowth.^[^ ^57^, ^58^, ^59^ ^]^ The pores created here during freeze‐drying are small (less than 20 µm), and are therefore unlikely to facilitate rapid cell invasion.^[^ ^60^ ^]^ Instead, they are more likely to aid in cell attachment by increasing the surface area and facilitating the physical adhesion between the ELR matrix and the cells.^[^ ^61^ ^]^ This in turn could promote the formation of a confluent endothelial monolayer.^[^ ^62^, ^63^ ^]^ A porous structure could also potentially improve the recruitment of blood‐borne cells, particularly monocytes and endothelial progenitor cells, which can differentiate into functional endothelial cells under the right biochemical cues.^[^ ^64^, ^65^ ^]^ The increase in cell attachment combined with the proteolytic degradability of the ELRs might also support cell integration, allowing for faster remodeling and vascularization of the ELR matrix. A material´s porous structure might also alter its blood compatibility, as reported previously for other materials,^[^ ^66^ ^]^ or influence the mechanical properties of the scaffold, which is why this was extensively addressed in the mechanical experiments.

The mechanical integrity of vascular grafts is one of the key factors for their functionality. The initial strain‐shift that was observed during the cyclic tensile testing between preconditioning and reaching a stable point (Figure 5), is likely due to accommodation of the ELR polymer chains and textile structure within the samples. This accommodation would include a straightening and tightening of the textile structure, as reported previously for other textiles^[^ ^67^ ^]^ as well as a rearrangement of polymer chains in reaction to the cyclic strain (Figure S4, Supporting Information), as has been observed for other polymers.^[^ ^68^ ^]^ Such a shift should be considered before in vivo implantation, to ensure that the graft diameter matches the dimensions of the target site after the accommodation took place. The results also showed that the nonreinforced ELR samples, independent of treatment, exhibited only minimal strain‐shift during the cyclic testing (Figure 5b, right panel), as expected due to the intrinsically elastic nature of the material, and the capability of our cross‐linking approach to translate such elasticity from the molecular level to the macrolevel, as previously reported by us.^[^ ^46^ ^]^ Importantly, cyclic stretching does not result in softening of the grafts, as indicated by the Young's modulus staying constant (i.e., slopes of the stress–strain curves remain equivalent). The results also show that textile‐reinforced grafts displayed nonlinear elastic properties, regardless of the specific treatment applied. This non‐Hookean behavior closely resembles the mechanical characteristics of native blood vessels. It is an important characteristic of native blood vessels that leads to the typical compliance behavior, with a higher compliance at lower pressure and, vice versa, lower compliance at higher pressure, which plays an important role for pulsatile flow transmission in arteries.^[^ ^69^ ^]^

The analysis of the stress/strain curves obtained during the tensile tests showed changes in the Young's modulus for higher pressure ranges depending on the treatment (Figure 6b, left panel). Specifically, it showed a reduction of the Young's modulus for the ECPD and LYO groups as well as an increase for the ACPD group in comparison to the Untreated group. This reduction indicates a softening of the grafts, possibly caused by a loosening of the connection between textile and ELR matrix because of the pores and bubbles introduced by these methods. As for the ACPD samples, one possible cause for the heightened Young's modulus could be an increase in crystallinity of the PET yarns, as scCO_2_ treatment of PET yarns has previously been reported to increase the crystallinity of the material leading to tougher and more rigid mechanical behavior.^[^ ^70^, ^71^, ^72^ ^]^ Another reason could be the compaction of the ELR matrix around the yarns, indicated by the reduced wall thickness, leading to more resistance against deformation, which would align with the observations discussed in the morphological evaluation of the grafts. Overall, the Young's modulus of all samples (Figure 6b, right panel) was below that of the human saphenous vein (2.25 MPa)^[^ ^73^ ^]^ or internal mammary artery (8.0 MPa),^[^ ^74^ ^]^ the current gold standard for small caliber vascular therapy. The observed differences may confer potential advantages for our grafts, as some studies suggest that prosthesis with lower Young's moduli exhibit superior performance compared to their stiffer counterparts.^[^ ^75^ ^]^ Additionally, postimplantation extracellular matrix remodeling, particularly collagen deposition, frequently results in increased graft stiffness.^[^ ^76^ ^]^ Therefore, the initial lower stiffness of the graft material may compensate for the effects of postimplantation stiffening, maintaining more favorable mechanical properties over time.

Following the cyclic testing to assess the elastic behavior after drying, substantially higher break stresses could be observed for the reinforced samples than for their nonreinforced counterparts (Figure 7). This difference is clearly due to the textile, as the ELRs on their own exhibit significantly lower break stresses, regardless of the treatment applied (Figure 7, right panel). Therefore, neither the textile reinforcement nor the mechanical integrity of the ELR matrix is impaired by any of the applied post‐treatments. These are very promising results, as the obtained values compare well with the circumferential break stress reported for the human saphenous vein (3.7 ± 2.0 MPa)^[^ ^73^ ^]^ and the internal mammary artery (4.1 MPa).^[^ ^74^ ^]^

Another essential characteristic that any vascular grafts should meet is sufficient suture retention, to ensure that the prosthesis stays attached to the native vasculature (anastomosis) without leakage or rupture. Overall, all the tested groups showed suture retention values well‐above those of native vessels (e.g., coronary arteries (2.01 ± 0.44 N), human internal mammary arteries (1.4 N), and saphenous veins (1.8 N)) (Figure 8).^[^ ^77^, ^78^ ^]^ The main component anchoring the suture line in the graft is the warp‐knitted PET mesh, and therefore the increase in suture retention observed for CPD samples is probably due to changes in this component. Indeed, scCO_2_ treatment has been reported to increase PET crystallinity, as discussed previously, which would correlate with the observed increase in suture retention. Combined with the positive outcomes from the anastomosis and perfusion tests (Figure S2 and Video S1, Supporting Information) these findings demonstrate suitable suturability.

Regardless of the applied treatment, the compliance behavior of the grafts closely resembles that reported for the human saphenous vein (21.0% ± 11.0% mmHg^−1^ × 10^−2^ at 30 mmHg mean pressure and 1.5% ± 0.4% mmHg^−1^ × 10^−2^ at 100 mmHg mean pressure) as well as that of native arteries (19.3% ± 11.9% mmHg^−1^ × 10^−2^ at 30 mmHg mean pressure, 2.6 ± 0.8% mmHg^−1^ × 10^−2^ at 100 mmHg mean pressure) (Figure 9b).^[^ ^79^ ^]^ Specifically, the values for the grafts lie between that of the artery and the vein, which present a good compromise for compliance matching when used as a arteriovenous shunt. This biomimicry of high compliance at low pressure and decreasing compliance with increasing pressure is essential to allow energy‐efficient transmission of the pulsatile blood flow.^[^ ^41^, ^80^ ^]^ Notably, commercial grafts made from ePTFE or Dacron usually exhibit constant compliance values over the whole physiological pressure range (1.6 and 1.9, respectively),^[^ ^69^ ^]^ which correlates with stenosis and graft failure.^[^ ^9^, ^79^ ^]^ Therefore, the combination of elastic ELR matrix and nonelastic PET fibers is highly effective in mimicking the behavior of native blood vessels, where under low pressure the load is predominantly borne by elastin components with high distensibility while toward higher pressure this load is transferred to the nonelastic collagen fibers.^[^ ^81^, ^82^ ^]^ While LYO had no apparent effect on the compliance behavior, both CPD approaches lead to an increase in compliance for lower pressures (Figure 9b). The increase in variability on the compliance values for ECPD samples when compared to the other groups, correlates with the visible defect formation and introduction of inhomogeneities within the ELR matrix during the ECPD process. Such defects are probably the reason for ECPD samples to burst during long‐term compliance testing. The changes observed over time during long‐term compliance testing for the rest of the treated groups (Figure 9c) suggest material rearrangements, and appear to mirror those seen in tensile testing, but at a slower rate. This difference in pace can be attributed to the distinct testing conditions. Specifically, during the tensile testing, the ends of the ring samples are free to move, allowing for more rapid rearrangement of the textile reinforcement. In contrast, during the compliance testing, both ends of the graft were fixed, which restricts the textile reinforcement's ability to rearrange and thereby slows down the accommodation of polymeric chains. It is important to consider this phenomenon during the implant design, and before in vivo implantation, to ensure that the final compliance of the graft matches that of the target implantation site.

Compliance mismatch can create highly turbulent flow conditions as well as high stress to the tissue, due to the big difference in stiffness, leading to intima hyperplasia, low patency, and finally graft failure.^[^ ^6^, ^83^, ^84^, ^85^ ^]^ So, achieving native‐like compliance in the implantable device is indispensable for clinical translation and was therefore one of our main points of interest. The generated knowledge about the impact between the drying scheme and the compliance behavior enables to match the intended compliance even after post‐treatment. The compliance of native vessels is site‐specific, and the optimal compliance of a cell‐free vascular graft is still being debated because mechanical properties of a graft may change due to in situ remodeling, potentially influencing the compliance behavior. In vivo studies are needed to fill the knowledge gap regarding the extent to which scaffold degradation can counterbalance the increase in stiffness due to collagen deposition.^[^ ^76^, ^86^, ^87^, ^88^ ^]^ These studies should be conducted using implants stored and sterilized in the same way as those planned for human application. Therefore, the knowledge about the effects of postprocessing gained through this work builds an important foundation for the successful clinical translation of these grafts.

Besides compliance and suture retention, burst pressure is a crucial indicator of mechanical integrity and reliability under physiological conditions, and it is therefore essential to elucidate the impact of the graft drying method on this variable. Regardless of treatment, the burst pressure of the grafts was 40–46% of the maximum burst strength of the human saphenous vein reported in literature (2250 mmHg) (Figure 10, left panel).^[^ ^89^ ^]^ However, the grafts will mostly be exposed to pressures in the 80–120 mmHg range. Therefore, the burst pressure of the developed graft is well‐above (10 times higher) the physiological arterial pressures and comparable to that reported for ePTFE grafts commonly used in clinics (600–1323 mmHg).^[^ ^90^, ^91^ ^]^ Indeed, recent studies have shown that grafts with burst strength values significantly lower than that of native blood vessels at the intended implantation site can still be safely implanted.^[^ ^88^, ^92^ ^]^

Related to cellular interaction, the ELR matrix's capacity to support endothelial cell attachment is crucial in tissue engineering, due to the significant role that these cells play in regulating vascular tone, inflammation, blood clotting, and angiogenesis.^[^ ^93^ ^]^ The cytocompatibility and bioactivity of ELRs has been extensively studied in previous works,^[^ ^20^, ^46^, ^48^, ^94^ ^]^ but so far the effects of the drying method on cellular attachment have not been studied. The only study that elucidates the effects of post treatments on ELRs demonstrates that autoclaving is an effective way to sterilize ELR structures.^[^ ^46^ ^]^ Unfortunately this method cannot always be applied for biohybrid constructs, considering the presence of additional material components other than ELRs. Upon autoclaving, the product remains in a hydrated state. In the case of products containing hydrolytically degradable components, this can increase the likelihood of their physical degradation, compromising their intended properties and functionality. Additionally, a wet state can complicate storage by increasing the risk of bacterial growth.^[^ ^28^, ^29^, ^30^, ^31^, ^32^ ^]^ Our study demonstrates that the implant's ability to facilitate cell attachment remains intact even after drying and rehydration. This indicates that the grafts can be stored in a dry state without losing this essential functionality. A dry prothesis also allows for the application of a wider range of sterilization techniques, while guaranteeing an effective, reproducible, and safe process.^[^ ^32^ ^]^

## 5 Conclusion

In our biohybrid graft presented here, we have refined the textile design, reduced the wall thickness and removed the macroporosity compared to our previous versions. All these improvements have been materialized in a vascular graft able to match the native compliance without the need for additional components (like an e‐spun layer) that can render the production process complex. Following refinement of the production technique, which led to greatly improved reproducibility, we performed a systematic study concerning the influence of different drying methods on graft performance. Through that, we demonstrated that both morphological and mechanical characteristics of the biohybrid scaffold can be altered to varying degrees depending on the applied method without affecting cell attachment. Our work thereby highlights the importance of establishing suitable drying schemes for a potential medical device early on during its development. Among the methods evaluated, ACPD enables the highest preservation when compared to the other drying schemes. Drying under appropriate conditions promises easy long‐term storage and opens the door to terminal sterilization of the finished product. Combined with reproducible production, we have made significant progress in overcoming some of the challenges associated with translating the textile‐reinforced ELR graft concept. These factors make the ELR‐based vascular graft a promising candidate for translation into clinic with the potential to significantly improve the quality of life for patients affected by cardiovascular disease and end‐stage renal disease.

## Conflict of Interest

The authors declare no conflict of interest.

## Supporting information



Supporting Information

Supplemental Video 1

Supplemental Video 2

## Data Availability

The data that support the findings of this study are available from the corresponding author upon reasonable request.
