# Supplementary material for: Biohybrid Vascular Graft Made of Textile‐Reinforced Elastin‐Like Recombinamers and Its Preservation via Drying Processes
Source: Adv Healthc Mater. 2025 May 3;14(15):2500482. doi: 10.1002/adhm.202500482 (PMC12147985; doi:10.1002/adhm.202500482)
Supplement: Supplementary file 1 — Supporting Information [file ADHM-14-0-s003.pdf]

ADVANCED HEALTHCARE MATERIALS

Supporting Information

for *Adv. Healthcare Mater.*, DOI 10.1002/adhm.202500482

Biohybrid Vascular Graft Made of Textile-Reinforced Elastin-Like Recombinamers and Its Preservation via Drying Processes

*Dominic Pascal Andre, Stephan Ruetten, José Carlos Rodríguez-Cabello, Stefan Jockenhoevel, Thomas Schmitz-Rode and Alicia Fernández-Colino**

Supporting Information

Biohybrid vascular graft made of textile-reinforced elastin-like recombinamers and its preservation via drying processes

*Dominic Pascal Andre[1], Stephan Ruetten[2], José Carlos Rodríguez-Cabello[3], Stefan Jockenhoevel[1], Thomas Schmitz-Rode[4], Alicia Fernández-Colino[1]**

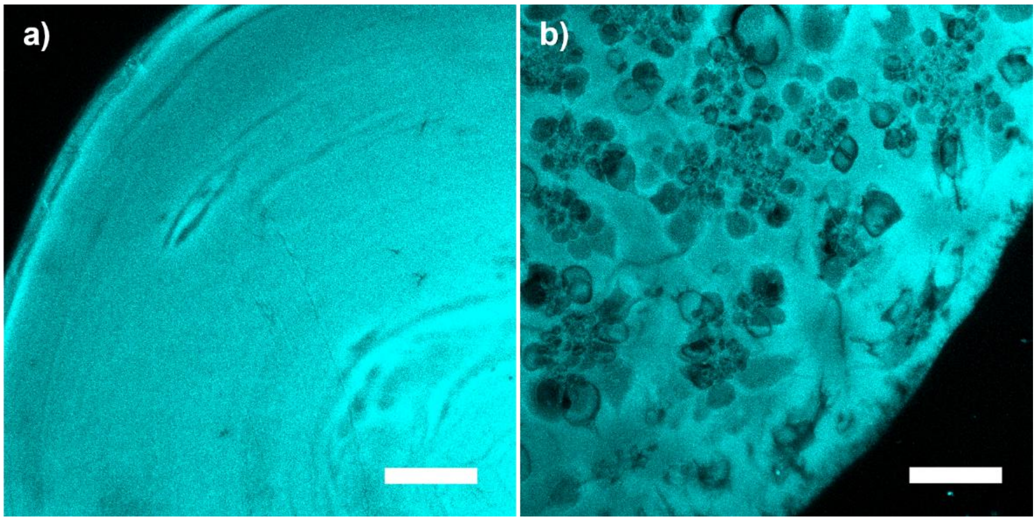

Figure S1. (a) ELR untreated in PBS under confocal microscope with no visible porosity. (b) ELR after lyophilization and rehydration in PBS under confocal microscope, showing persisting porosity. Scale bars: 100µm

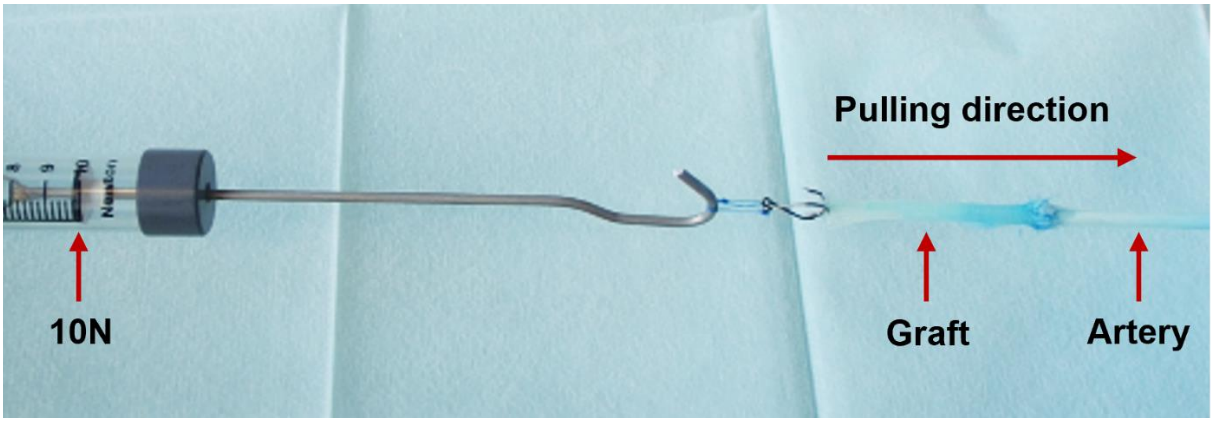

Figure S2. Testing of suture strength between porcine artery and biohybrid graft by manual pulling using an analog 10N force meter connected to the graft by two hooks.

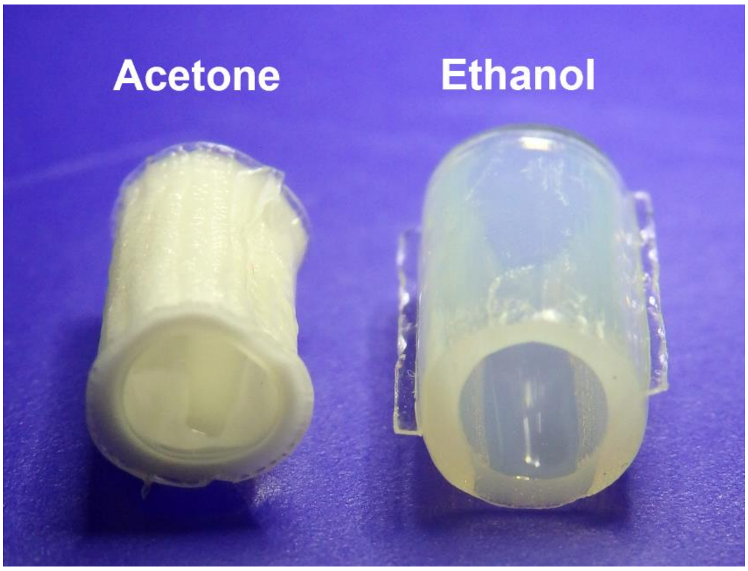

Figure S3. Non-reinforced ELR-grafts immersed in either 100 % acetone or 100% ethanol overnight. Pictures were taken before drying and show that acetone leads to a partial collapse of the matrix while in 100% ethanol, the configuration is similar to that in PBS at room temperature.

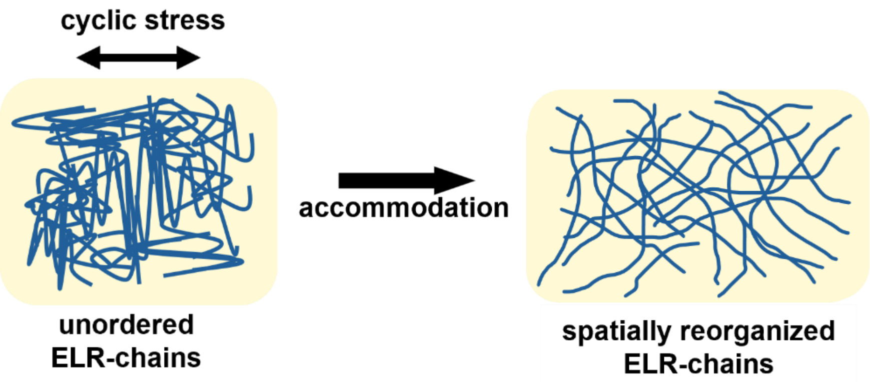

Figure S4. Schematic of the accommodation behavior of the ELR-chains under cyclic-stress. Reorganization of the polymer chains along the pulling axis leads to a dilatation of the matrix, leading to a strain-shift..
